# Design of Glycerol-Based Solvents for the Immobilization of Palladium Nanocatalysts: A Hydrogenation Study

**DOI:** 10.1021/acssuschemeng.1c01694

**Published:** 2021-04-30

**Authors:** Alejandro Leal-Duaso, Isabelle Favier, Daniel Pla, Elísabet Pires, Montserrat Gómez

**Affiliations:** † Department of Organic Chemistry, Faculty of Science, University of Zaragoza, Calle Pedro Cerbuna, 12, E-50009 Zaragoza, Spain; ‡ Instituto de Síntesis Química y Catálisis Homogénea (ISQCH−CSIC). Faculty of Science, University of Zaragoza, Pedro Cerbuna, 12, E-50009 Zaragoza, Spain; § Laboratoire Hétérochimie Fondamentale et Appliquée, UMR CNRS 5069, Université de Toulouse 3 − Paul Sabatier, 118 Route de Narbonne, F-31062 Toulouse Cedex 9, France

**Keywords:** Green solvents, palladium nanoparticles, glycerol ethers, DES, catalysis, hydrogenation

## Abstract

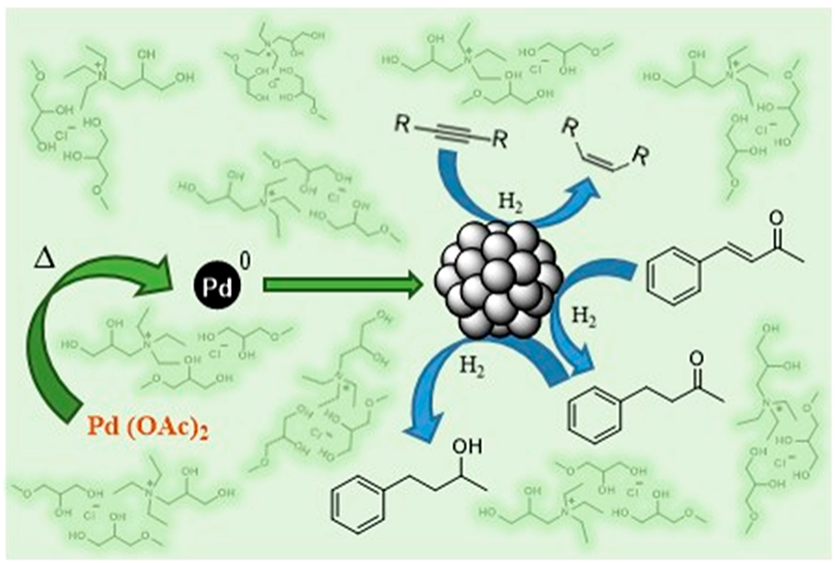

Twenty-one green solvents, including glycerol-derived ethers, and their eutectic mixtures with two renewable ammonium salts, were used for the straightforward synthesis, stabilization, and immobilization of palladium nanoparticles (Pd NPs). The nature of the solvent allows tuning of the characteristics and properties of resulting catalytic systems in terms of particle size and morphology, stability, reactivity, and recoverability. Pd NPs immobilized in glycerol-based solvents were applied in the catalytic hydrogenation of alkenes, alkynes, and carbonyl compounds, as well as in the selective semihydrogenation of alkynes to alkenes. The optimal experimental parameters and the influence on the reactivity of the physicochemical properties of solvent, mainly the viscosity, were studied. Moreover, the most active and recoverable catalytic system, **Pd NPs/N00Cl-100**, was fully characterized both in the liquid phase and in the solid state, and its deactivation upon recovery was analyzed.

## Abstract 2

Glyceryl monoether-derived deep eutectic solvents exhibit adequate properties for the preparation, immobilization, and stabilization of highly active and recoverable Pd-NP catalytic systems for hydrogenation reactions of a wide scope of substrates.

## Introduction

Metallic nanocatalysts have attracted huge interest in fields such as nanomaterials, optics, catalysis, and biomedicine, due to their electronic properties and high specific surface areas.^1^ Among all the transition metals, palladium has been one of the most applied in the synthesis of nanoparticles (NPs),^2^ offering interesting morphologies for catalytic purposes.^3−5^ In contrast to top-down methodologies that achieve the split of the metal by physical means, bottom-up approaches starting from metal salts and organometallic precursors allow a better control of the size, morphology, and stability of the NPs in a reproducible way.^6^ However, NPs are unstable clusters that tend to agglomerate, increasing their size and reducing their active surface. For this reason, the enhancement of their stability is a key aspect that can be kinetically achieved by introducing steric, electronic, or electrosteric repulsion forces.^7^ Organic ligands, surfactants, and polymers have been widely used for NPs stabilization.^8,9^ Once stabilized, NPs can be used directly or supported, blending the main features of both homogeneous and heterogeneous catalysts.^10^ The immobilization, either on solids or liquids, facilitates the recovery and reuse of metal NPs, and above all, increases their stability.^11^ Apart from solid supports such as clays, zeolites, oxides, polymers, and carbonaceous materials,^9,12,13^ structurally organized liquid phases are being successfully applied for the immobilization of NPs.^14,15^

In the past decade, the development of green solvents has enabled their intensive application owing to advantageous physicochemical properties such as both moderated volatility and flammability, biodegradability and low eco-toxicity.^16^ Among them, biobased solvents such as polyols,^17^ water,^4^ ionic liquids (ILs),^18^ deep eutectic solvents (DESs),^19^ and supercritical fluids^4^ have already been used for the synthesis of NPs. In particular, glycerol has shown valuable properties for the preparation and stabilization of metallic species,^20^ due to its hydrogen-bond supramolecular structure.^15,21^ On account of the current bioglycerine surplus,^22^ a plethora of DESs have been described using glycerol as a hydrogen-bond donor (HBD), in combination with different ammonium salts such as choline chloride (ChCl).^23,24^ Some glycerol derivatives, including carbonates, ketals, esters, and ethers, have also been studied as useful and versatile biosolvents.^22,25^ In fact, green methods for the synthesis of monoethers and diethers of glycerol with multiple substitution patterns have been recently described.^26−30^ Thanks to their interesting physicochemical properties and their very low eco-toxicity,^31^ these glycerol-derived solvents are being used in novel applications,^32^ as chemical precursors,^33^ and also for replacing glycerol as the HBD component in eutectic solvents.^34^ Nevertheless, none of them has been used as a solvent for the synthesis of NPs, and only for the immobilization of preformed Pd NPs in a very recent work.^35^

Metal-catalyzed hydrogenation reactions of unsaturated compounds represent efficient and sustainable procedures, due to its total atom economy and low waste generation. Since the first Ni-catalyzed hydrogenation reactions described by Sabatier and Senderens,^36^ palladium, platinum, and nickel catalysts have been profusely applied in industrial hydrogenations for the large-scale production of different petrochemicals, foods, drugs, and fertilizers.^37−39^ In this context, the use of metal nanoparticles immobilized onto solid supports has demonstrated their interest, in particular for recycling purposes.^1^ However, liquid phases have been less considered as supports in the immobilization of NPs for catalytic applications.^4,17,40^

To date, metal NPs in different ILs have been efficiently used in selective hydrogenations.^41,42^ In this case, the IL acts as the reaction medium, as well as the NP stabilizer, ligand, and support.^18^ Moreover, the selectivity can be modulated by changing the structure of the solvent.^18^ Despite their chemical similarity to ILs, DESs have been barely applied in hydrogenation reactions. Just a few works have appeared, such as the hydrogenation of methyl cinnamate in urea-carbohydrate mixtures using the Wilkinson’s catalyst,^43^ or the use of Pd NPs immobilized in choline-based DESs in turn microencapsulated in polysilanes for the hydrogenation of unsaturated compounds.^44^ Finally, Gómez and co-workers have used polyols, mainly glycerol, as media for the immobilization of Ni NPs, Pd NPs, and bimetallic Pd/Cu NPs for hydrogenation processes.^17,39,45,46^

In this work, we study the utility of glycerol-derived solvents such as mono-, di-, and triethers of glycerol, as well as eutectic mixtures thereof, both as media for the sustainable synthesis, stabilization, and immobilization of Pd NPs and also for the selective hydrogenation of different unsaturated substrates. Morphology, stability, reactivity, and recoverability of the catalytic systems have been analyzed, discussing the relevance of the nature of the solvent.

## Experimental Section

The chemicals used in this work as reagent grade are listed in the Supporting Information (SI). All the manipulations were performed using Schlenk techniques under an argon atmosphere, unless otherwise stated. High-pressure reactions were carried out in a Top Industrie Autoclave. Conversions and yields were determined by gas chromatography coupled to mass spectrometry (GC-MS), using a PerkinElmer Clarus 500 chromatograph equipped with a flame ionization detector (FID), and a PerkinElmer Clarus MS 560 spectrometer as the mass detector. GC experimental conditions are detailed in the SI.

Glycerol-based solvents, catalytic systems, and reaction products were fully characterized. Experimental and characterization details are gathered in the SI.

### Synthesis of the Glycerol-Based Solvents

Glycerol **R00** monoethers were selectively prepared from glycidol and the corresponding alcohol by means of basic catalysis, following our previously described methodology.^27^ Glyceryl symmetric **R0R** diethers and nonsymmetric **R0R′** ones were synthesized starting from epichlorohydrin and the corresponding alcohol or alcohols, according to our previous works.^29,30^ Glycerol **RRR** triethers were obtained by methylation of the glycerol diether or monoether (1 mol) with iodomethane (1.4 mol), prior to ether deprotonation with NaH (1.2 mol) in dry THF (150 mL). After heating at 60 °C for 30–90 min, the reaction mixture was poured into cold water (133 mL) and quenched with HCl (0.2 mL, 0.3 M). Glycerol triethers were extracted with diethyl ether (3 × 66 mL), and the combined organic extracts were washed with an aqueous solution of sodium thiosulfate (5 wt %) to eliminate iodine traces. Then, the organic phase was dried over MgSO_4_ and filtered off, and the diethyl ether was removed under reduced pressure to furnish the desired solvents. All the glycerol-derived solvents were completely purified by vacuum distillation and dried at 80 °C overnight under vacuum prior to use.

### Synthesis of the Pd Nanoparticles Immobilized in Glycerol Ethers

In a Fisher–Porter bottle, 11.2 mg (0.05 mmol) of Pd(OAc)_2_ and 111 mg of poly-*N*-vinylpyrrolidone (PVP, 10 000 g·mol^–1^) were completely dissolved, under an argon atmosphere, in 5 mL of the glycerol ether dried as described above. That system was placed under vacuum, pressurized with hydrogen (3 bar), and stirred at 80 °C for 10 h. Then, the resulting black colloidal solution (10 mM of Pd NPs) was cannulated and kept under argon prior to use in catalysis.

### Synthesis of the Pd NPs Immobilized in Glycerol-Based DES

In a Schlenk flask, 11.2 mg (0.05 mmol) of Pd(OAc)_2_ were dissolved in 5 mL of the dried glycerol-based DES (**ChCl-HBD**, **N00Cl-HBD**) and stirred under argon at 80 °C for 10 h. The black colloidal suspension obtained (10 mM or 1 mol % Pd) was kept under argon.

### Isolation of the Pd NPs in the Solid State

After the above-described synthesis, NPs in glycerol-derived solvents were transferred to a centrifugation tube and ethanol (5 mL) was added. Centrifugation was carried out at 3000 rpm for 5 min, and then the solution was separated by decantation. This process was repeated twice with ethanol (5 mL), followed by two additional washings with acetone (2 mL). The remaining black powder was dried under vacuum at 80 °C overnight. Elemental analysis (Pd determined by ICP-AES): Pd 88.9%, C 5.29%, N 0.95%, H 0.46% (in **N00Cl-100** solvent).

### General Procedure for Pd-Catalyzed Hydrogenation Reactions

The corresponding substrate (1 mmol) and the suspension of Pd NPs/glycerol-derived solvent (0.01–1 mol % Pd, 1 mL) were placed into a Fisher–Porter bottle (for hydrogenations working from 1 to 3 bar total pressure) or in an autoclave vessel (for hydrogenations working from 10 to 55 bar pressure) and stirred under an argon atmosphere. The reaction mixture was put under vacuum and pressurized with hydrogen at the desired pressure. The system was stirred and heated at 80 °C in a silicone oil bath for the specified reaction times. Then, the reaction system was cooled down to room temperature and then depressurized. The organic compounds were extracted with *n*-pentane (5 × 5 mL) at 40 °C using vigorous stirring and periods of 15 min between each extraction. Purification of products was carried out by column chromatography or Kugelrohr distillation. Upon extraction of the organic products, the catalytic system was dried at 80 °C under vacuum for 5 h prior to a new run.

## Results and Discussion

### Selection and Synthesis of the Glycerol-Derived Solvents

As we have previously described, a great variety of differently substituted glycerol ethers can be synthesized in an eco-friendly manner in order to achieve a portfolio of renewable solvents with interesting physicochemical properties.^27,34^ Some features of these green media such as their low eco-toxicity, low volatility, viscosity, and high solubilizing ability for organic and inorganic compounds^34^ encouraged us to use them for the synthesis of original catalytic systems based on palladium nanoparticles immobilized in liquid phases (**Pd NPs/solvent**). With this aim, the properties of the glycerol-derived medium not only allow the reduction of the palladium salt used as a zerovalent Pd NPs precursor owing to the solubilization of H_2_ but also enable the formation and stabilization of the metal NPs. Given the importance of Pd nanocatalysts toward surface-assisted reactions,^17^ we decided to apply the **Pd NPs/solvent** systems in different hydrogenation catalytic processes. In addition, the solubility of organic substrates and the recovery of reaction products from the glycerol-based reaction medium was also assessed in terms of simple manipulation and high reproducibility.

**Scheme 1:**
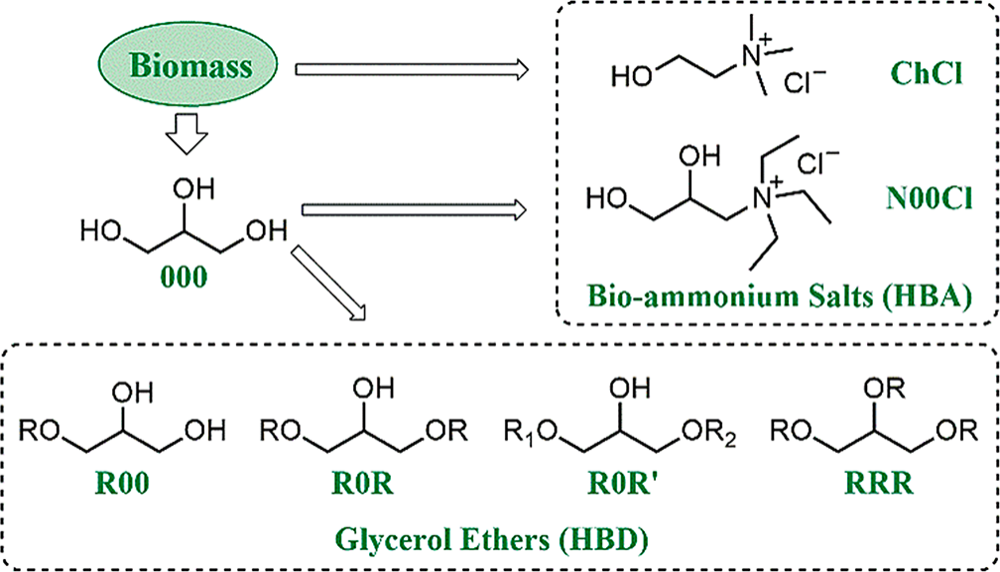
Bioammonium Salts (Hydrogen Bond Acceptors, HBA) and Different Kinds of Glycerol Ethers (Hydrogen Bond Donors, HBD) Used in This Work

**Figure 1:**
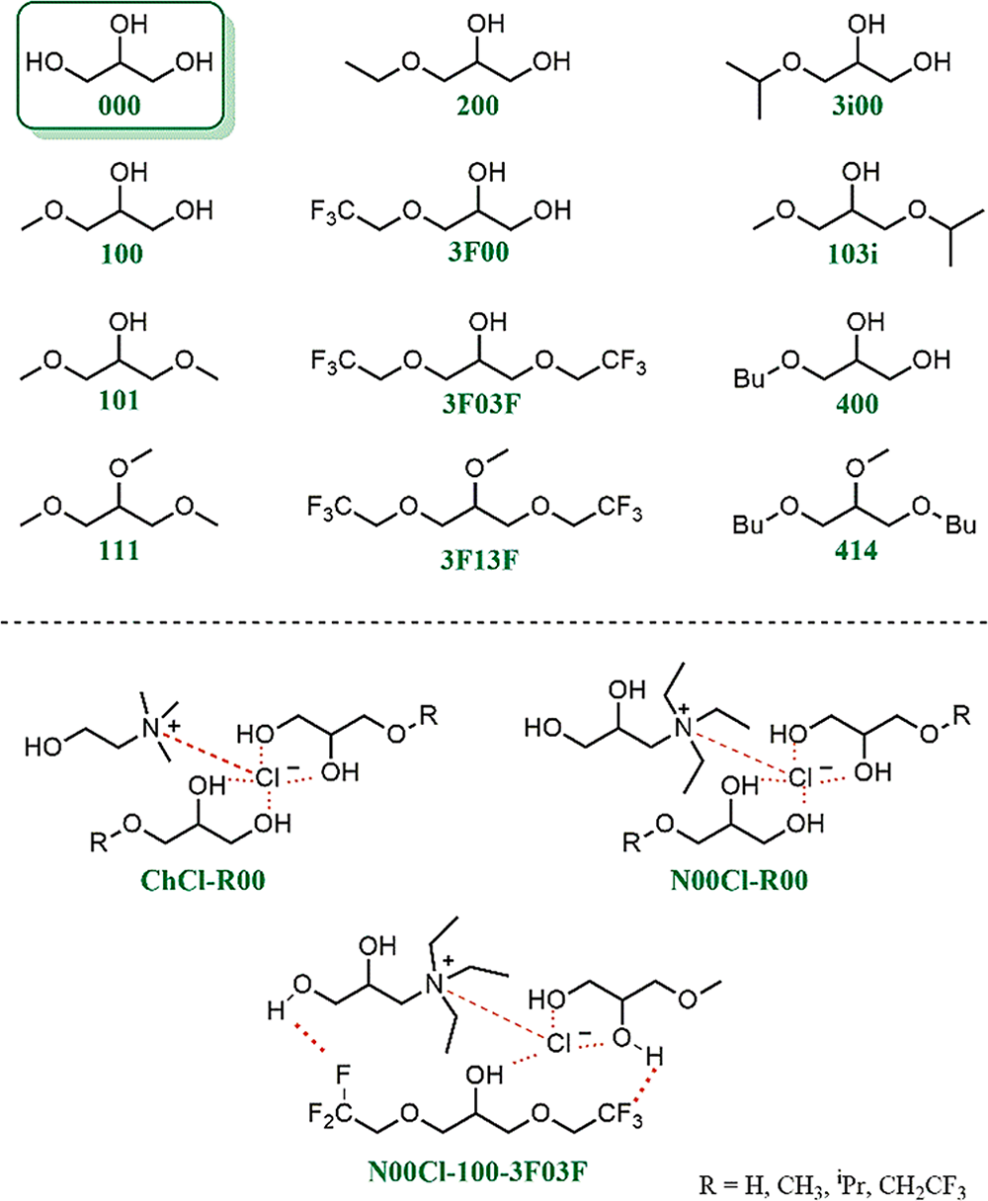
Selection of glycerol-derived solvents for the synthesis of immobilized Pd NPs and their application in catalysis. For eutectic solvents (**ChCl-R00**, **N00Cl-R00**, **N00Cl-100–3F03F**), plausible intermolecular interactions are highlighted in orange.

Taking all these aspects into consideration, a selection of different glycerol-derived biosolvents is reported herein. As shown in the Scheme 1, glycerol (**000**), glyceryl monoethers (**R00**), diethers (symmetric **R0R** and nonsymmetric **R0R′**), and triethers (**RRR**), as well as their eutectic mixtures with two biobased ammonium salts, namely, choline chloride (**ChCl**) and *N*,*N*,*N*-triethyl-2,3-dihydroxy-propan-1-aminium chloride (**N00Cl**, see Scheme 1), were chosen. With this purpose, glycerol ethers bearing different long, branched, and fluorinated **R** alkyl chains were synthesized. More specifically, the selected substituents were methyl groups in compounds **100**, **101**, **103i**, and **111**; an ethyl group in monoether **200**; a 2,2,2-trifluoroethyl chain in compounds **3F00**, **3F03F**, and **3F13F**; an isopropyl chain in compounds **3i00** and **103i**; and a butyl chain in compounds **400** and **414** (see Figure 1).

On the other hand, for the preparation of the eutectic solvents, glycerol (**000**) and the glyceryl monoethers **100**, **3i00**, and **3F00** were used as the HBD component in combination with the two above-mentioned salts, **ChCl** and **N00Cl**, as the HBA component, according to a 1:2 HBA/HBD molar ratio. Also, the ternary mixture **N00Cl-100-3F03F**, including the symmetric diether **3F03F**, was prepared in a 1:1:1 molar ratio (see Figure 1).

### Synthesis and Characterization of the Pd NPs Immobilized in Glycerol Ethers

In this work, Pd NPs were synthesized by the reduction of a palladium precursor, Pd(OAc)_2_, in the solvent also acting as the immobilization liquid support. Glycerol (**000**), glycerol monoethers (**100**, **200**, **3F00**, **3i00**, **400**), glyceryl diethers (**101**, **103i**, **3F03F**), and glycerol triethers (**111**, **3F13F**, **414**) as solvents, as well as PVP as the stabilizer of the nanoparticles, were used.

**Scheme 2:**
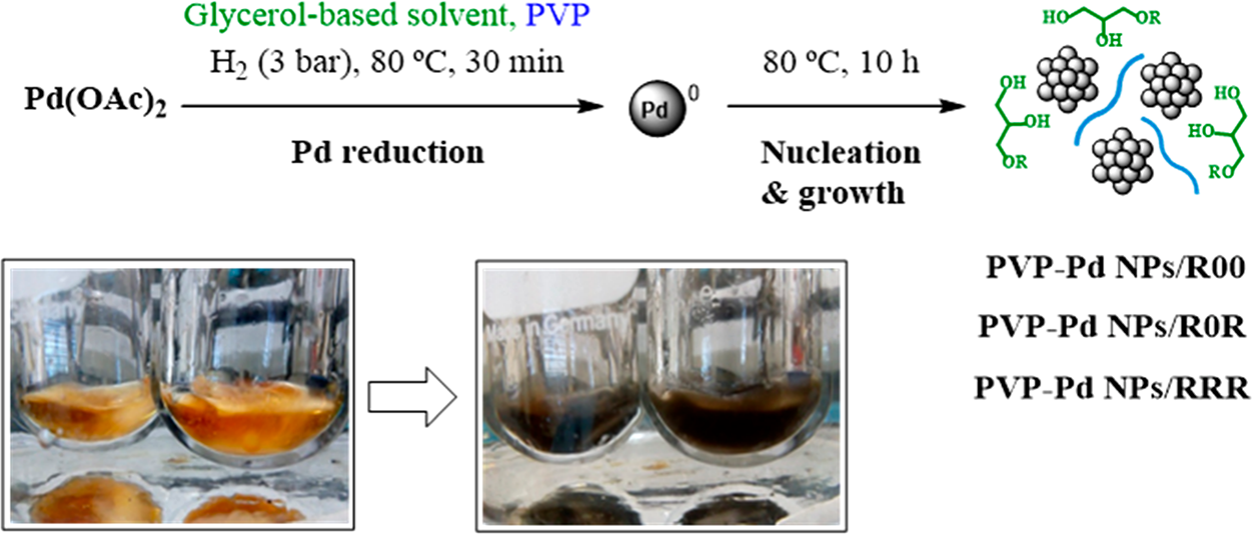
Synthesis of PVP-Pd NPs Immobilized in Glycerol Ethers

Under mild conditions, at a hydrogen pressure of 3 bar and a temperature of 80 °C, the reduction of Pd(II) could be evidenced in the glycerol-derived media after 30 min, thanks to a color change of the resulting suspension, from orange to Pd(0) black (Scheme 2). In order to guarantee the full Pd reduction and optimal nucleation and growth of the PVP-Pd NPs, a standard reaction time of 10 h was applied. In all the cases, using this synthetic procedure, black Pd NP homogeneous suspensions with a high stability over time were formed. It is noteworthy to mention that, in these media, the use of molecular hydrogen permits the palladium reduction, avoiding the oxidation of the glycerol-derived solvent.^17,20^

**Figure 2:**
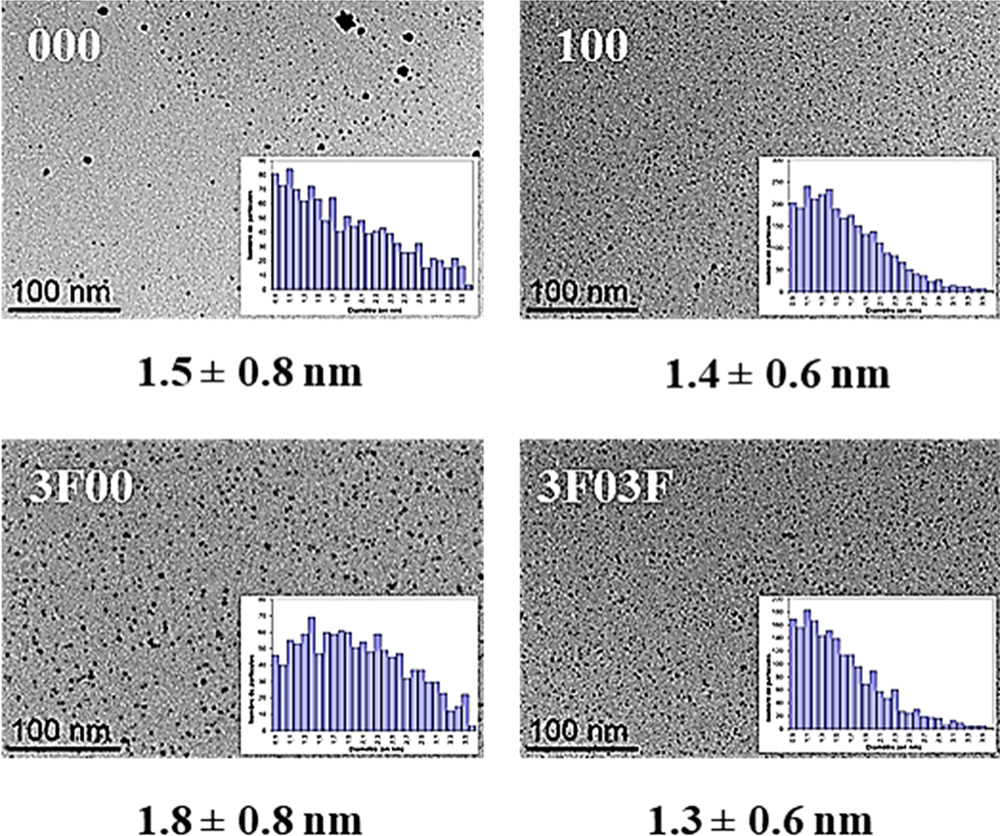
TEM micrographs and size distributions of PVP-Pd NPs immobilized in glycerol (**000**) and in three selected glycerol-derived solvents: **100**, **3F00**, and **3F03F**.

Transmission electron microscopy (TEM) analyses of these colloidal suspensions in glycerol ethers revealed spherical-shaped and well-dispersed Pd NPs (Figure 2). However, in the case of pure glycerol (**000**), the dispersion and homogeneity of the NPs were limited by its high viscosity (1200 cP versus 0.7–68 cP for the studied glycerol ethers at room temperature). All TEM micrographs and size distributions are detailed in the Supporting Information, except for solvents **101** and **111**, which could not be recorded in liquid media due to the higher volatility of both solvents. Interestingly, the amount and mean diameter of NPs showed a direct dependence on the nature of the glycerol-derived solvent used in the synthesis. In general, NPs prepared in more polar and viscous media increased their particle size. As it can be observed in Figure 2, along the sequence of polarity **3F03F** < **100** < **3F00**, the mean particle size was respectively 1.3, 1.4, and 1.8 nm, albeit with relatively large size distributions. When comparing the influence of the polarity and hydrogen bond ability of the solvent on the morphology of the synthesized NPs, using two differently substituted glycerol ethers such as **3F00** and **3F03F**, it can be observed that the higher polarity and HBD ability of the monoether seems to be related to the formation of NPs presenting higher mean sizes. Other aspects with minor influence on polarity, such as the ramification or the length of the ether alkyl chain, did not seem to have a significant influence on the NPs’ shape or size. For instance, in the cases of using **200** and **3i00**, with a similar polarity, both solvents provided similar sized Pd NPs (1.5 ± 0.7 nm).

### Synthesis and Characterization of the Pd NPs Immobilized in Glycerol-Derived DES

**Scheme 3:**
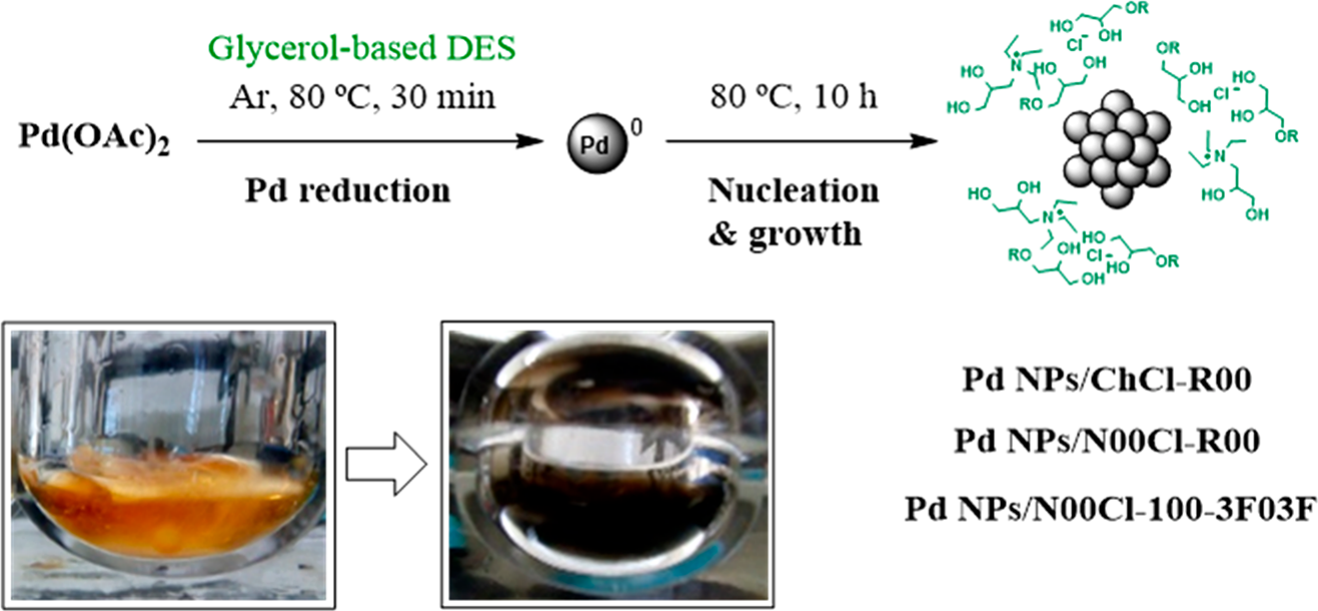
Synthesis of PVP-Free Pd NPs Immobilized in Glycerol-Derived DES

The synthesis of Pd NPs in the glycerol-derived DES, using the same palladium precursor, was achieved by heating the mixture at 80 °C under an argon atmosphere in the absence of any additional stabilizers (Scheme 3). The ionic character and supramolecular arrangement via hydrogen bond in the eutectic solvents provided the NP stabilization, the DES acting both as a liquid support and as an electrosteric stabilizer.

**Figure 3:**
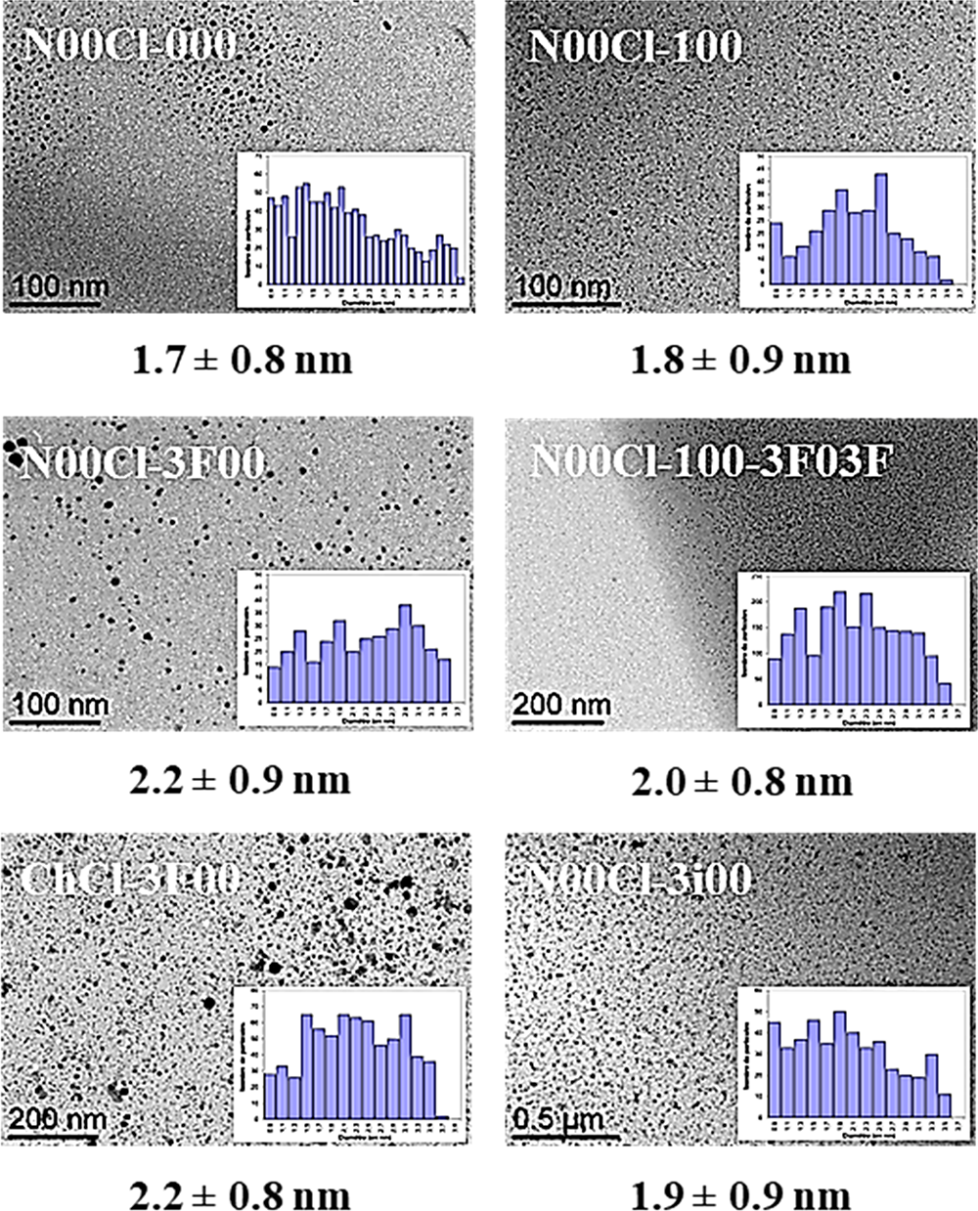
TEM micrographs of Pd NPs immobilized in six glycerol-derived eutectic solvents: **N00Cl-000**, **N00Cl-100**, **N00Cl-3F00**, **N00Cl-100–3F03F**, **ChCl-3F00**, and **N00Cl-3i00**, with their corresponding size distribution diagrams.

As it has been recently described, the small amounts of water present in glycerol or in these hygroscopic solvents would be responsible for the reduction of palladium(II) into zerovalent palladium species.^46−48^ The resulting Pd NP black colloidal suspensions obtained using this straightforward procedure were stable. In particular, NPs synthesized in these glycerol-derived eutectic media showed different morphologies, populations, and size distributions than in the case of using glycerol ethers (Figure 3). It can be observed that the nature of the medium has a bigger influence on the morphology of the synthesized Pd NPs, compared to the case of using glyceryl ethers. Hence, the selection of the solvent becomes an interesting tool for the design of metal NPs.

Pd NPs synthesized in fluorinated media, such as **N00Cl-3F00** and **ChCl-3F00**, presented bigger mean diameters (*ca*. 2.2 nm) than in the case of the less polar solvents, *i*.*e.*, 1.8 ± 0.9 nm for **N00Cl-100** and 1.9 ± 0.9 nm for **N00Cl-3i00**. In addition, the high viscosity of the solvent also influenced the Pd NPs dispersion. An inhomogeneous distribution of nanoparticles in solvents **N00Cl-000** and **N00Cl-3F00**, with viscosity values of 2693 and 553 cP, respectively, was observed. Although similar particle diameters and size distributions were found for Pd NPs synthesized in DES composed of the same glyceryl ether and different HBA salt, an influence of the nature of the ammonium salt on the NPs’ stability was identified. Thus, the presence of **ChCl** instead of **N00Cl** triggered some palladium agglomeration in the case of solvents **ChCl-000**, **ChCl-3i00**, and **ChCl-3F00**. Nevertheless, when using the fluorinated ether **3F00**, similar trends were observed with both ammonium salts. Moreover, Pd NPs in **N00Cl-100-3F03F** showed a good dispersion of homogeneous particles with an average size of 2.0 ± 0.8 nm. The interest in these ternary mixture systems is based on the possibility of fine-tuning the characteristics of metal NPs for specific catalytic applications. As expected, the size was intermediate between those of **N00Cl-100** and **N00Cl-3F00**, as a consequence of the intermediate property values of this medium.

**Figure 4:**
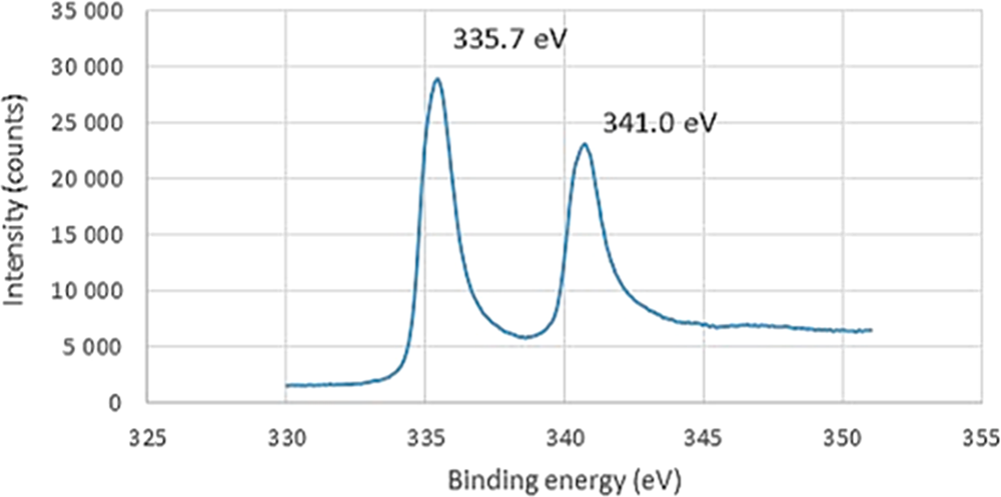
High resolution XPS spectrum of **Pd NPs/N00Cl-100** in the solid state for the Pd(0) region.

In order to evidence the structure of these immobilized Pd NPs, **Pd NPs/N00Cl-100** was chosen as a homogeneously dispersed nanoparticle suspension in a DES for full characterization both in the liquid phase and in the solid state (isolated by centrifugation from the corresponding colloidal solution). FT-IR analysis of Pd NPs in the solid state suggested the presence of remaining solvent **N00Cl-100** in the isolated NPs (see Figure S21), as a result of the strong interaction between the ionic solvent and palladium during the Pd NPs synthesis, thus demonstrating the major role of DES as an electrosteric stabilizer. Both elemental and ICP-AES analyses showed a consistent palladium percentage (*ca*. 89%). As could be expected, the rest of the mass seems to correspond to the above-mentioned solvent **N00Cl-100** stabilizing the NPs. As shown in Figure 4, an X-ray photoelectronic spectroscopy (XPS) survey spectrum evidenced that palladium was in a zerovalent oxidation state (experimental values: 335.7 and 341.0 eV versus 335.4 and 340.3 eV for bulk Pd(0) 3d_5/2_ and 3d_3/2_ peaks, respectively).^49^ Furthermore, the XPS analysis revealed a N/Cl ratio of 3:8 (see Table S1 in the SI). This fact could be explained by the partial degradation of the ammonium salt of DES (**N00Cl**), in the form of volatile amines (Hofmann elimination), with X-ray irradiation.

**Figure 5:**
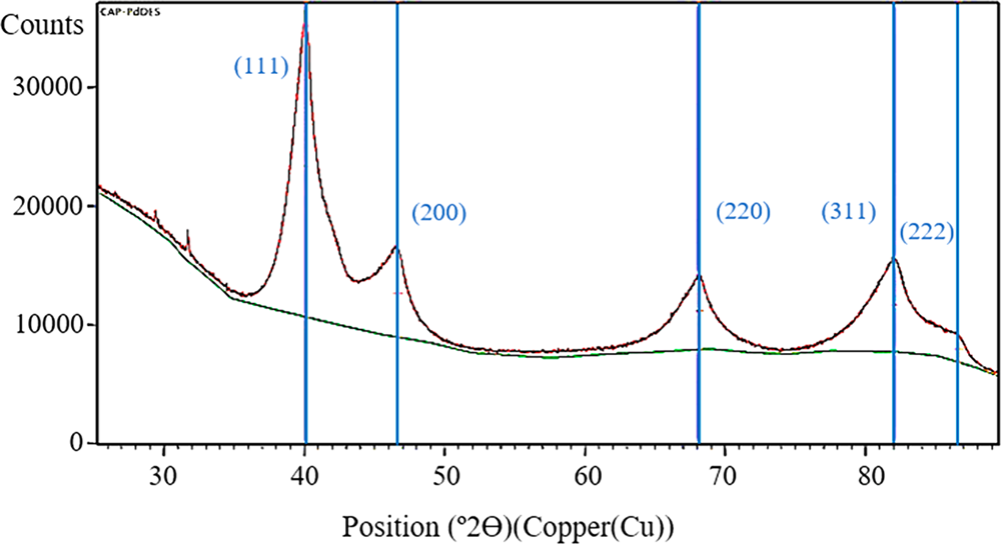
Powder X-ray diffractogram of **Pd NPs/N00Cl-100**, showing the diffraction pattern of fcc Pd(0) (blue lines).

Powder X-ray diffraction (PXRD) analysis allowed calculation of a crystallite size of 3.9 ± 0.9 nm for Pd(0), which was consistent with the mean diameter of 3.3 ± 2.1 nm calculated in the solid state using TEM microscopy (see Figure S19 and Figure S22 in the SI). The five main crystallographic diffraction planes of crystalline Pd(0) can be appreciated in Figure 5 (blue lines). Experimental crystallographic data fit the face centered cubic structure of Pd NPs.^46^

### Pd-Catalyzed Hydrogenation Reactions

**Scheme 4:**
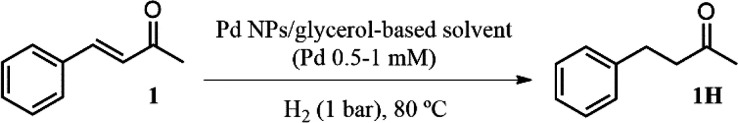
Benchmark Hydrogenation Reaction of (*E*)-4-Phenylbut-3-en-2-one (**1**), Catalyzed by Pd NPs Immobilized in Glycerol-Based Solvents

Pd NPs immobilized in the glycerol-based biosolvents, including glycerol, glyceryl ethers, and their derived DESs, were applied in the catalytic hydrogenation of different functional groups. Experimental parameters were optimized for the chosen benchmark hydrogenation of (*E*)-4-phenylbut-3-en-2-one (1, Scheme 4). Among them, catalyst amount, hydrogen pressure, temperature, reaction time, and solvent volume were considered.

Table 1 gathers the conversions of (*E*)-4-phenylbut-3-en-2-one (**1**) catalyzed by Pd NPs immobilized both in glycerol (**000**, Table 1, entries 1–5) and in glycerol monoether **100** (Table 1, entries 6–10). For all the reactions, the only product obtained was 4-phenylbutan-2-one (1H), with isolated yields from 75% to 92% when full conversions were reached, by extraction with *n*-pentane as described in the Experimental Section. As can be seen, the mildest reaction conditions affording the complete hydrogenation of the substrate in glycerol were obtained using a 0.1 mol % palladium loading and 1 bar of H_2_ pressure, at 80 °C for 2 h (Table 1, entry 4). Lower palladium loadings, temperature, and reaction time provided partial conversions (Table 1, entries 1–3, 6, and 7). Interestingly, in the case of using the system **Pd NPs/100**, total conversion and selectivity could be achieved with a lower palladium loading (0.05 mol % or 0.5 mM Pd). For this reaction medium, the best experimental conditions were 1 bar of H_2_ and heating at 80 °C for 2 h (Table 1, entry 8).

**Table 1:** Influence of the Reaction Conditions in the Hydrogenation of (*E*)-4-Phenylbut-3-en-2-one Catalyzed by Pd NPs Immobilized in Glycerol (**000**) and **100**

entry^a^	Pd load (mol %)	time (h)	conversion (%)^b^	TON (TOF, h^–1^)
**Pd NPs/000**				
**1**	0.05	0.5	50	1000 (2000)
**2**	0.1	0.5	75	750 (1500)
**3**	0.1	1	92	920 (920)
**4**	0.1	2	100	1000 (500)
**5**	0.5	0.5	100	200 (400)
**Pd NPs/100**				
**6**^c^	0.05	2	81	1620 (810)
**7**	0.05	0.5	58	1160 (2320)
**8**	0.05	2	100	2000 (1000)
**9**	0.1	0.5	100	1000 (2000)
**10**	0.5	0.5	100	200 (400)

a Reaction conditions: The substrate (1 mmol) was dissolved in the colloidal solution (1 mL) of preformed Pd NPs in glycerol (**000**) or **100** solvent in a Fisher-Porter vessel. The vessel was placed under vacuum prior to its filling with H_2_ (1 bar), sealed, and heated at 80 °C for 30 min unless otherwise stated. The reaction crudes were extracted using *n*-pentane.

b Conversion was determined in duplicate by GC and NMR using *n*-decane as an internal standard. Selectivity was 100% for all the reactions.

c Reaction carried out at 60 °C.

Therefore, Pd NPs immobilized in glycerol monoether **100** showed better activity than in glycerol. Slight activity differences in terms of catalytic turnover number and frequency could be observed between NPs immobilized in glycerol and **100** (TON 1000 vs 1160; TOF 2000 h^–1^ vs 2320 h^–1^; Table 1, entries 1 and 7, respectively). This can be attributed to the higher population of smaller Pd NPs in the case of using the glycerol ether, which probably would increase the total active surface palladium and hence the catalytic activity.

The influence of the reaction solvent was also assessed in order to determine the most active Pd NPs/solvent system. Thus, we evaluated the catalytic activity at a shorter reaction time (30 min, see Table 2). Also, catalytic systems were recovered after the reaction and reused in a second run with the aim of evaluating their stability. In the first run, full conversions were achieved in only 30 min of reaction time when using Pd NPs immobilized in glycerol ethers, both monoethers **200**, **3i00**, and **3F00** (Table 2, entries 3–5) and diether **3F03F** (Table 2, entry 6). When comparing Pd NPs/DES systems, NPs immobilized in mixtures of glycerol ethers with **N00Cl** (Table 2, entries 11–14) led to better yields than mixtures with **ChCl** (Table 2, entries 8 and 9). In the case of using glycerol (Table 2, entry 1) and mixtures of ethers with **ChCl**, conversions not higher than 50% were observed. In addition, Pd NPs suspended in mixtures of glycerol with both ammonium salts (**ChCl-000** and **N00Cl-000**, Table 2, entries 7 and 10, respectively) provided worse results than using their respective glycerol ether mixtures (**ChCl-R00** and **N00Cl-R00**). In terms of recycling, only **Pd NPs/N00Cl-100** gave excellent conversion and selectivity in the second run. Thus, this system showed the highest TON and TOF values (3920, 3920 h^–1^). For the rest of the catalytic systems, conversions did not exceed 43% in the second run.

**Table 2:** Hydrogenation of (*E*)-4-Phenylbut-3-en-2-one (**1**) Catalyzed by Pd NPs Immobilized in Different Glycerol-Based Solvents (Two Runs Are Shown)

entry^a^	solvent	conv. (%)^b^ (1st/2nd run)	yield (%)^c^ (1st/2nd run)	accumulated TON^d^
**1**	**000**	50/31	30/22	1620
**2**	**100**	58/12	46/8	1400
**3**	**200**	100/43	67/26	2460
**4**	**3i00**	100/18	80/17	2360
**5**	**3F00**	100/14	94/7	2280
**6**	**3F03F**	100/36	91/29	2720
**7**	**ChCl-000**	22/4	15/5	520
**8**	**ChCl-100**	49/9	28/3	1160
**9**	**ChCl-3F00**	30/10	20/6	800
**10**	**N00Cl-000**	29/16	20/12	900
**11**	**N00Cl-100**	97/99	82/94	3920
**12**	**N00Cl-3i00**	71/26	60/23	1940
**13**	**N00Cl-3F00**	62/32	52/26	1880
**14**	**N00Cl-100-3F03F**	97/40	90/34	2740

a Reaction conditions: The substrate (1 mmol) was dissolved in the colloidal solution of Pd NPs/glycerol-based solvent (1 mL, 0.5 mM Pd, 0.05 mol %), prior to the pressurization with 1 bar of H_2_ heating at 80 °C for 30 min.

b Conversion was determined by GC-MS and NMR using *n*-decane as a standard. Selectivity was 100% in all the cases except with **200** (86%).

c Isolated yields.

d Accumulated TON in two runs is the same value than average TOF in h^–1^.

The chemical nature of the solvent determines its capacity of solvating reagents, probably having an effect on the reaction manifolds. In this case, no solubility limitations of the organic compounds are considered a priori, as both glycerol ethers and their derived eutectic solvents display a wide range of polarity and hydrophobicity values.^32,34^ It is also interesting to remark on the full selectivity observed with all of these systems, precluding the reduction of carbonyl groups under mild reaction conditions. On top of that, the hydrogen bond network in the studied solvents guarantees the solvation of the palladium species involved in the catalytic process.^35^ To our knowledge, no information about the solubility of H_2_ in either of the glycerol-derived solvents is available, with the exception of pure glycerol.^15^ Due to the high reactivities observed with these catalytic systems, the solubility of H_2_ seems not to be a limiting parameter in the case of the studied solvents.

**Figure 6:**
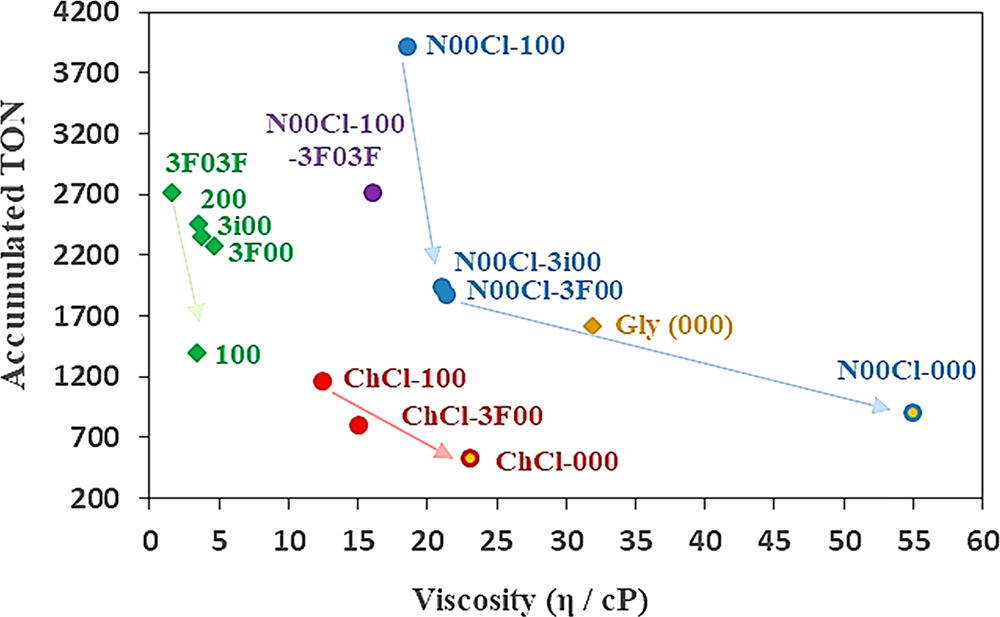
Relationship between the catalytic activity (accumulated TON in two cycles) of the systems **Pd NPs/solvent** in the benchmark hydrogenation reaction and the dynamic viscosity of the solvent (in cPs or mPa·s at 80 °C).

In a previous work, it was demonstrated that the high viscosity of glycerol (with a dynamic viscosity of 31.9 cP at 80 °C) hinders the diffusion of both the reagents and catalyst.^35,48^ This can be also a key point in order to explain the reactivity diminution observed in the hydrogenation reaction of substrate **1** from the glycerol ether systems (displaying a viscosity of 1.6–4.7 cP at 80 °C) to glyceryl ether-derived DES (12.5–21.4 cP at 80 °C), and even more for the DES composed of pure glycerol (23.1 and 55 cP for **ChCl-000** and **N00Cl-000**, respectively, at 80 °C).^34^ The representation of the accumulated TON values versus the dynamic viscosity of the solvent (at the reaction temperature, 80 °C) in Figure 6 evidences this effect for each family of glycerol-derived media.

For instance, along the growing viscosity sequence **N00Cl-100** < **N00Cl-3i00** < **N00Cl-3F00** < **N00Cl-000**, the inverse trend in the catalytic productivity (3920 > 1940 > 1880 > 900) can be observed. A similar behavior in the case of mixtures with **ChCl** and in the case of glycerylethers can be also implemented. Other physicochemical properties such as density, surface tension, refractivity, or heat capacity of the solvent have also been considered.^34^ In the case of surface tension, a weak relationship with reactivity was found, probably due to the same reasons above explained for viscosity.

In addition to the properties of the reaction medium, the size and morphology of Pd NPs directly impact the catalytic reactivity.^9^ Therefore, viscosity differences are not enough to explain catalytic differences, for example the highest reactivity for Pd NPs immobilized in **N00Cl-100** and **N00Cl-100–3F03F**, with respect to monoethers such as **100**. Thus, we observed that systems with a higher number of smaller NPs (mean diameter around 1–2 nm) presented enhanced reactivity, as in the case of **Pd NPs/3F03F**, due to a larger active surface. Conversely, systems with less-spherical and bigger NPs, dispersed in an inhomogeneous manner, such as **ChCl-3F00** or **N00Cl-3F00**, would present less total active surface with respect to **N00Cl-100**, in agreement with a decrease in the TON. In addition, the formation of agglomerates notably reduces the catalytic active surface, which was probably the case for **ChCl-000** and **ChCl-3F00** solvents. Thus, Pd NPs’ morphology is modified by fine-tuning of the solvent, triggering an effect on catalytic reactivity. For example, the size of NPs in **N00Cl-100-3F03F** in between those of **N00Cl-100** and **N00Cl-3F00** resulted in an intermediate TON value (2740), with respect to those of **N00Cl-100** (3920) and **N00Cl-3F00** (1880). Furthermore, the observed catalytic behavior also indicates that the DES supramolecular structure impacts the size of PdNPs (effect on the PdNPs formation mechanism) and dispersion (effect on stabilization), **N00Cl-100** being the best solvent to lead to small PdNPs and avoid their agglomeration, even after catalysis. On the basis of reported studies related to nanoparticles/solvent interactions, the electronic state of the Pd NPs in the different solvents should be quite similar.^50,51^

**Figure 7:**
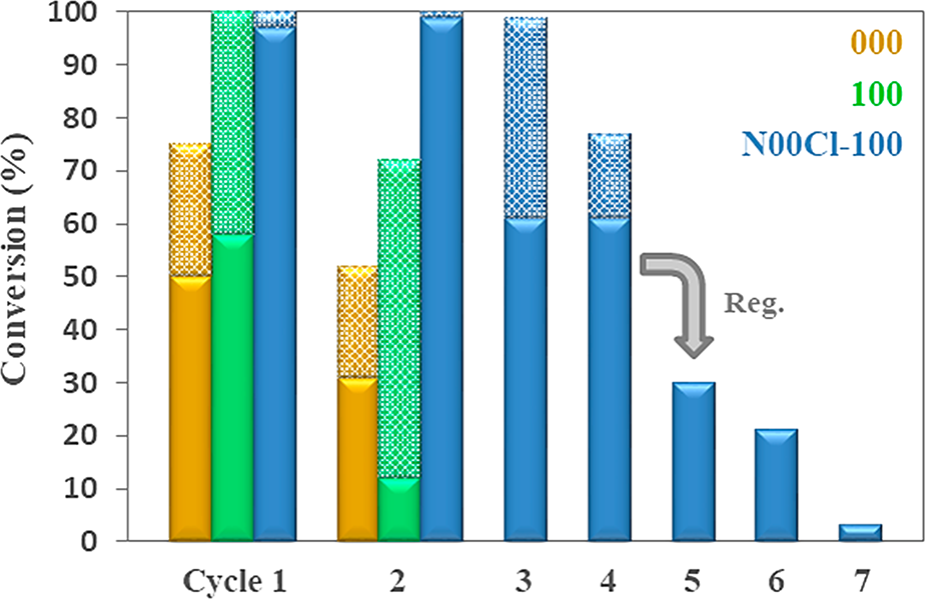
Conversions obtained after 30 min in the benchmark reaction catalyzed by Pd NPs immobilized in **000** (in yellow), **100** (in green), and **N00Cl-100** (in blue), upon recovery using two Pd loads (0.1 mol %, dashed; 0.05 mol %, solid).

Furthermore, the solvent giving the best catalytic results, **N00Cl-100**, was used to perform additional recycling studies for the benchmark hydrogenation reaction (Scheme 4). With this aim, the recyclability of the system **Pd NPs/N00Cl-100**, compared to **Pd NPs/000** and **Pd NPs/100**, was evaluated. Results gathered in Figure 7 showed that, using 0.1 mol % Pd, the deactivation of Pd NPs immobilized in glycerol (**000**) or in the monoether **100** started in the second cycle. In the case of using the system **Pd NPs/N00Cl-100**, quantitative conversions were achieved for three consecutive cycles. Notably, Pd NPs suspended in the eutectic medium displayed greater stability and recyclability than in the cases of the monoether or pure glycerol, even using a lower palladium load.

**Figure 8:**
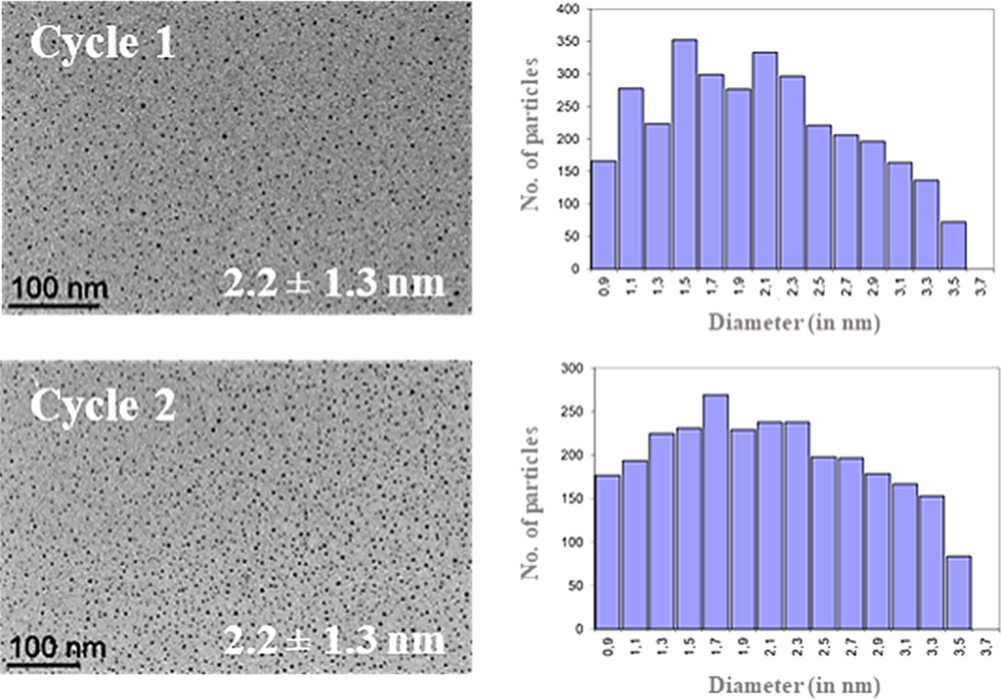
TEM images and size distribution diagrams of **Pd NPs/N00Cl-100** after catalyzing one and two runs of the benchmark hydrogenation reaction (see Scheme 4).

When using 0.05 mol % Pd (solid bars in Figure 7), the system **Pd NPs/N00Cl-100** was reused in two reaction cycles with quantitative conversions and two additional runs with 61% of conversion. Unfortunately, cycles 5–7 showed a progressive deactivation of the catalytic system. Attempts to regenerate the catalyst, by hydrogen and thermal treatment, or by the addition of solvent, were not successful. TEM analysis of the reused Pd NPs immobilized in **N00Cl-100** (Figure 8) showed no modifications in NP size and morphology after two catalytic cycles. In addition, Pd NP dispersion and size distribution diagrams were maintained. Above this, ICP-AES analyses of the crude extracts revealed the absence of palladium (<0.05 ppm, detection limit for palladium). This information points to a surface reactivity mechanism. According to the Ostwald ripening, the leaching of atoms during the reaction and further redeposition on the surface of the NPs would progressively increase the NPs size, but this was not observed in our study. The additional stabilization of NPs by the solvent would reinforce the heterogeneous mechanism, as described for glycerol.^15^ However, from the fifth run onward, some palladium agglomeration was visually observed. This issue is the possible reason for the progressive NPs deactivation.

Additionally, the solvent stability during the recycling was studied by ^1^H NMR analysis of the recovered **N00Cl-100** after centrifugation of the catalytic phase, proving that no decomposition occurred (see Figure S23 in the SI). The as-recovered catalyst was redispersed in fresh **N00Cl-100** and used in the hydrogenation of (*E*)-4-phenylbut-3-en-2-one (**1**), exhibiting an important deactivation (<10% conversion). This behavior denotes a major effect of DESs on the stabilization of Pd NPs during their synthesis.

The most active and recoverable catalytic system, **Pd NPs/N00Cl-100**, was successfully applied in the hydrogenation of different compounds (Tables 3 and 4).

**Table 3:**
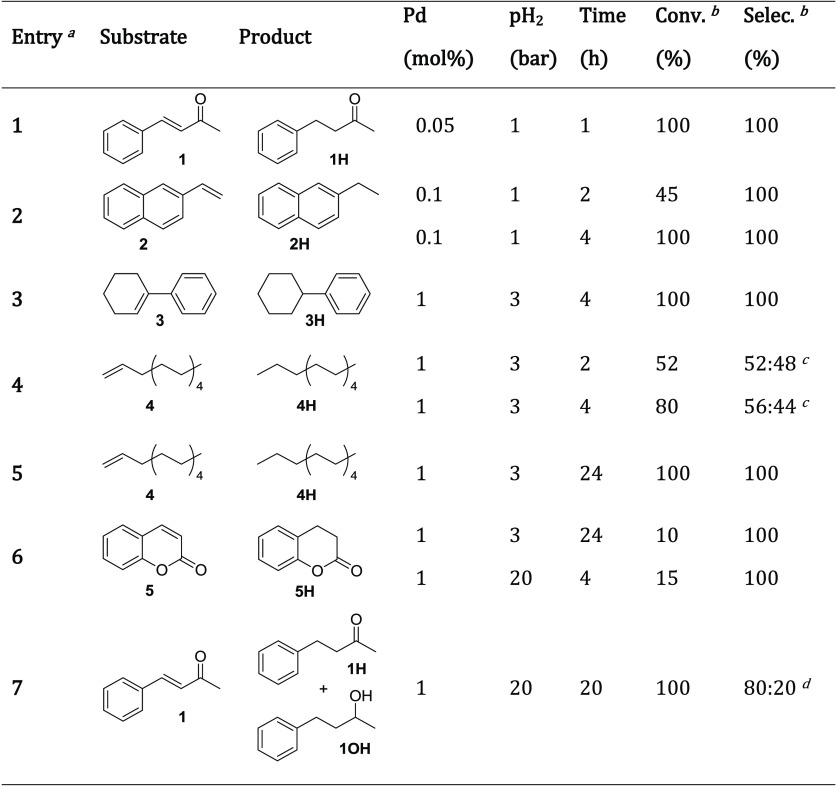
Hydrogenation Scope Catalyzed by **Pd NPs/N00Cl-100**

a Reaction conditions: The substrate (1 mmol) was dissolved in the colloidal solution of preformed Pd NPs in **N00Cl-100** (1 mL, 1–10 mM Pd, 0.1–1 mol % Pd) in a Fisher–Porter vessel. Then, the vessel was placed under vacuum prior to its filling with H_2_ (1–20 bar), sealed, and heated at 80 °C. Reaction crudes were extracted using *n*-pentane.

b Conversion and selectivity were determined by ^1^H NMR using 1,4-dioxane as a standard.

c **4H**/internal alkenes ratio.

d **1H**/**1OH** ratio.

**Table 4:**
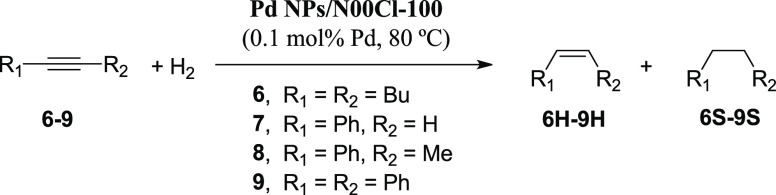
Hydrogenation of Alkynes Catalyzed by **Pd NPs/N00Cl-100**

entry^a^	substrate	pH_2_ (bar)	time (h)	conv.^b^ (%)	selectivity H/S^b^^,^^c^ (%)
**1**	**6**	3	2	100	99/1 (94)
**2**^d^	**6**	3	23	100	84/16 (56)
**3**^d^	**6**	3	72	100	0/100 (93)
**4**	**7**	1	0.13	41	100/0 (14)
**5**	**7**	1	0.25	81	100/0 (62)
**6**	**7**	1	0.50	100	62/38 (89)
**7**	**7**	3	1	100	2/98 (93)
**8**^d^	**8**	1	0.5	16	97/3 (14)
**9**^d^	**8**	3	20	100	0/100 (96)
**10**	**9**	1	0.5	0	0/0 (0)
**11**	**9**	1	2	>99	87/13 (87)
**12**	**9**	3	2	100	0/100 (96)

a Reaction conditions: The substrate (1 mmol) was dissolved in the colloidal solution of Pd NPs in **N00Cl-100** solvent (1 mL, 1 mM Pd, 0.1 mol % Pd), prior to the vessel pressurization with H_2_ (1–3 bar) and heating at 80 °C.

b Conversion and selectivity were determined by ^1^H NMR using 1,4-dioxane as internal standard.

c Isolated yields given in brackets.

d 1 mol % Pd.

The hydrogenation of conjugated alkenes, such as (*E*)-4-phenylbut-3-en-2-one (**1**), 2-vinylnaphthalene (**2**), and 2,3,4,5-tetrahydro-1,1′-biphenyl (**3**), was quantitatively achieved using low Pd loadings and mild reaction conditions (Table 3, entries 1–3). In the case of nonconjugated linear alkenes such as 1-dodecene (**4**), the hydrogenation product **4H** and different isomerization products were obtained at short reaction times (Table 3, entry 4). The complete hydrogenation of **4** toward *n*-dodecane (**4H**) was only achieved at longer reaction times (Table 3, entry 5), meaning that internal alkenes can also be successfully hydrogenated. However, this catalyst was not capable of completing the hydrogenation of a highly deactivated olefin such as *2H*-chromen-2-one (**5**, entry 6). All these results point out that the catalytic activity of **Pd NPs/N00Cl-100** is at least comparable to that of other Pd NPs described in glycerol for similar substrates and reaction conditions.^45,46^ Additionally, this catalytic system required harsher reaction conditions in the reduction of ketones to the corresponding alcohols. Therefore, the hydrogenation of substrate **1** under 20 bar of H_2_ pressure afforded the total hydrogenation of the double bond as well as partial reduction of the carbonyl group (Table 3, entry 7).

### Pd-Catalyzed Semihydrogenation of Alkynes

The semihydrogenation of alkynes to alkenes, and in particular, phenyl acetylene to styrene, is a key enabling transformation for polymer industries as unwanted traces of alkynes in feedstock components act as poison for the polymerization catalysts used in polystyrene production plants.^52^ However, it is difficult to find the optimal catalyst and experimental conditions for carrying out this reaction avoiding the formation of the alkanes.^39^ We considered it interesting to apply the system **Pd NPs/N00Cl-100** to the semihydrogenation of different alkynes (Table 4).

In order to find the optimal reaction conditions to carry out the semihydrogenation of substrates **6**–**9**, different palladium loadings, hydrogen pressures, and reaction times were assessed. In the case of 5-decyne (**6**), **Pd NPs/N00Cl-100** provided its semihydrogenation with total conversion and selectivity toward (*Z)*-5-decene (**6H**, Table 4, entry 1), under mild conditions (0.1 mol % Pd, 3 bar H_2_, 2 h). However, at longer reaction times, full hydrogenation was observed (Table 4, entries 2–3). In the case of aryl alkynes such as phenyl acetylene (**7**) and 1-phenylpropyne (**8**), semihydrogenation toward **7H** and **8H** was not fully accomplished using this catalyst (Table 4, entries 4–6 and 8). Thus, these substrates were semihydrogenated with partial conversions (Table 4, entries 5 and 8). The best tested conditions in the hydrogenation reaction of diphenylacetylene (**9**) allowed completion of the conversion of the alkyne to an 87:13 9*H*/9S mixture (Table 4, entry 11). Therefore, the highest selectivity was achieved for internal alkyl alkynes.

In addition, the complete hydrogenation of the differently activated alkynes **6**–**9** was achieved by **Pd NPs/N00Cl-100** (Table 4, entries 3, 7, 9, and 12) using palladium loadings lower than 1 mol % and mild reaction conditions (3 bar H_2_, 80 °C).

Finally, the catalytic system **Pd NPs/N00Cl-100** was also studied toward hydrodehalogenation reactions of haloarenes (see Scheme S1 in the SI), exhibiting moderate to complete conversions, thus broadening the efficiency of this NP system in hydrogen-mediated catalytic processes.

## Conclusions

In this work, 21 green solvents such as glycerol and glyceryl monoethers, diethers, and triethers, as well as the eutectic solvents resulting from the mixture with two bioammonium salts (**ChCl** and **N00Cl**), have been effectively used for the straightforward synthesis, immobilization, and stabilization of palladium nanoparticles (Pd NPs). In the case of the DES, no additional stabilizers nor reductive agents are necessary for the reduction of the palladium precursor, Pd(OAc)_2_, thus improving the sustainability of Pd NPs preparation. The strong coordination of DES to Pd(0) has demonstrated the NP stabilization role of the solvent. The morphology of Pd NPs strongly depended on the physicochemical properties of the used solvent, thus allowing the tuning of size, distribution, and homogeneity of the NPs.

Palladium nanoparticles immobilized in glycerol-based solvents have shown high catalytic activity in the hydrogenation of conjugated and nonconjugated alkenes, alkynes, and carbonyl compounds, as well as relevant selectivity in the semihydrogenation of alkynes to alkenes. Best results are obtained in pure glyceryl ethers and in **N00Cl-R00** solvents, **N00Cl-100** being the best media. Finally, the physicochemical properties of glycerol-derived solvents as liquid supports for nanocatalysis play a key role in the activity outcomes and the recyclability of the catalytic systems.

## Supporting Information Available

Acronyms and abbreviations, chemicals and gas chromatography analysis, characterization of used glycerol-based solvents, characterization of catalytic systems (TEM analyses, full characterization of system **Pd NPs/N00Cl-100**), characterization of hydrogenation products, H_2_-mediated hydrodehalogenation results (PDF)

## Dedication

Dedicated to the memory of Prof. José Ignacio García Laureiro (1959–2019).
