# Supplementary material for: Design of Glycerol-Based Solvents for the Immobilization of Palladium Nanocatalysts: A Hydrogenation Study
Source: ACS Sustain Chem Eng. 2021 Apr 30;9(19):6875–85. doi: 10.1021/acssuschemeng.1c01694 (PMC8908245; doi:10.1021/acssuschemeng.1c01694)
Supplement: Supplementary file 1 — sc1c01694_si_001.pdf [file sc1c01694_si_001.pdf]

Electronic Supplementary Information

Design of glycerol-based solvents for the immobilization of palladium nanocatalysts: A hydrogenation study

Alejandro Leal-Duaso,[a],[b] *Isabelle Favier,*[c] *Daniel Pla,*[c] *Elísabet Pires,**[a],[b] *and Montserrat Gómez**[c]

a Department of Organic Chemistry, Faculty of Science, University of Zaragoza, calle Pedro Cerbuna, 12. E-50009, Zaragoza, Spain.

b Instituto de Síntesis Química y Catálisis Homogénea (ISQCH-CSIC). Faculty of Science, University of Zaragoza, calle Pedro Cerbuna, 12. E-50009 Zaragoza, Spain.

c Laboratoire Hétérochimie Fondamentale et Appliquée, UMR CNRS 5069, Université de Toulouse 3 – Paul Sabatier, 118 Route de Narbonne, F-31062 Toulouse Cedex 9, France.

**Elísabet Pires, e-mail:* [*epires@unizar.es*](mailto:epires@unizar.es)

**Montserrat Gómez, e-mail:* [*gomez@chimie.ups-tlse.fr*](mailto:gomez@chimie.ups-tlse.fr)

Number of pages: 20
Number of figures: 23
Number of tables: 1
Number of schemes : 1

Table of contents

1. - Acronyms and abbreviations S3

2. - Chemicals and gas chromatography analysis S4

3. - Characterization of used glycerol-based solvents S6

4. - Characterization of catalytic systems S10

4.1. - TEM analyses S10

4.2. - Full characterization of system Pd NPs/N00Cl-100 S13

4.3. – ^{1}H NMR spectra of DES after hydrogenation S17

5. - Characterization of hydrogenation products S18

6. - H_2-mediated hydrodehalogenation results S20

1. - Acronyms and abbreviations

**000**	Glycerol
**100**	3-Methoxy-1,2-propanediol
**101**	1,3-Dimethoxy-2-propanol
**103i**	1-Isopropoxy-3-methoxy-2-propanol
**111**	1,2,3-Trimethoxypropane
**112**	1-Ethoxy-2,3-dimethoxypropane
**200**	3-Ethoxy-1,2-propanediol
**212**	1,3-Diethoxy-2-methoxypropane
**300**	3-Propoxy-1,2-propanediol
**313**	1,3-Dipropoxy-2-methoxypropane
**3F00**	3-(2,2,2-Trifluoroethoxy)-1,2-propanediol
**3F03F**	1,3-Bis(2,2,2-trifluoroethoxy)-2-propanol
**3F13F**	2-Methoxy-1,3-bis(2,2,2-trifluoroethoxy)propane
**3i00**	3-Isopropoxy-1,2-propanediol
**400**	3-Butoxy-1,2-propanediol
**414**	1,3-Dibutoxy-2-methoxypropane

**η**	Dynamic viscosity
**b.p.**	Boiling point
**ChCl**	Choline chloride
**ChCl-100**	Mixture of **ChCl** and glycerol ether **100**
**$CDCl_3$**	Deuterated chloroform
**cP**	Centipoise (dynamic viscosity unit)
**DCM**	Dichloromethane
**DES**	Deep Eutectic Solvent
**DMSO-d_6**	Deuterated dimethyl sulfoxide
**GC**	Gas Chromatography
**GC-MS**	Gas Chromatography coupled to Mass Spectrometry
**HBA**	Hydrogen-Bond Acceptor
**HBD**	Hydrogen-Bond Donor
**IL**	Ionic Liquid
**IR**	Infrared spectroscopy
**N00Cl**	*N,N,N*-Triethyl-2,3-dihydroxypropan-1-aminium chloride
**N00Cl-100**	Mixture of **N00Cl** and glycerol ether **100**
**N00Cl-100-3F03F**	Ternary mixture of **N00Cl**, **100** and **3F03F**
**NPs**	Nanoparticles
**PVP**	Poly-*N*-vinyl-2-pyrrolidone
**PXRD**	Powder X-ray Diffraction
**R**	Ether alkyl chain
**R00**	3-Alkoxy-1,2-propanediols (glycerol monoethers)
**R0R**	1,3-Dialkoxy-2-propanols (symmetric glycerol diethers)
**R0R'**	1-Alkoxy-3-alkoxy'-2-propanols (non-symmetric glycerol diethers)
**RRR**	1,2,3-Trialkoxypropanes (glycerol triethers)
**TEM**	Transmission Electron Microscopy
**THF**	Tetrahydrofuran
**TOF**	Turnover Frequency
**TON**	Turnover Number
**XPS**	X-ray Photoelectronic Spectroscopy

2. – Chemicals and gas chromatography analysis

The chemicals used in this work, except metallic hydroxides, were purchased as reagent grade. Glycerol, glycidol, epichlorohydrin, 3-chloropropane-1,2-diol, palladium (II) acetate, poly-*N*-vinylpyrrolidone (PVP, with average Mw of 10000 g/mol), sodium hydride, iodomethane, *n*-decane, *n*-pentane and deuterated solvents were purchased from Sigma-Aldrich. 2,2,2-Trifluoroethanol (TFE), butanol and choline chloride were acquired from Alfa Aesar. Methanol, ethanol, isopropanol, KOH and NaOH were obtained from Scharlab. All the alcohols were dried and distilled over calcium hydride prior to use.

All the reactions were analyzed by Gas Chromatography coupled to Mass Spectrometry (GC-MS).

Gas chromatography analyses were carried out in a Perkin Elmer Clarus 500 chromatograph equipped with a flame ionization detector (FID), and using a 5% phenylmethylsiloxane SGE BPX5 column (30 m × 0.32 mm × 0.25 μm) and helium as carrier gas. Mass spectrometry was provided by a Perkin Elmer Clarus MS 560 spectrometer as the mass detector.

GC temperature program: injector temperature: 250 ºC; detector temperature: 250 ºC; GC temperature oven program: 45 º C starting temperature for 2 min, +20 ºC min^{-1} temperature gradient up to 300 ºC (for 12.8 min), and 300 ºC final isotherm for 5 min.

GC retention times: *n*-decane: 5.2 min; 4-phenylbutan-2-one: 7.5 min; *(E)*-4-phenylbut-3-en-2-one (**1**): 8.4 min; for other hydrogenation products see Section S5.

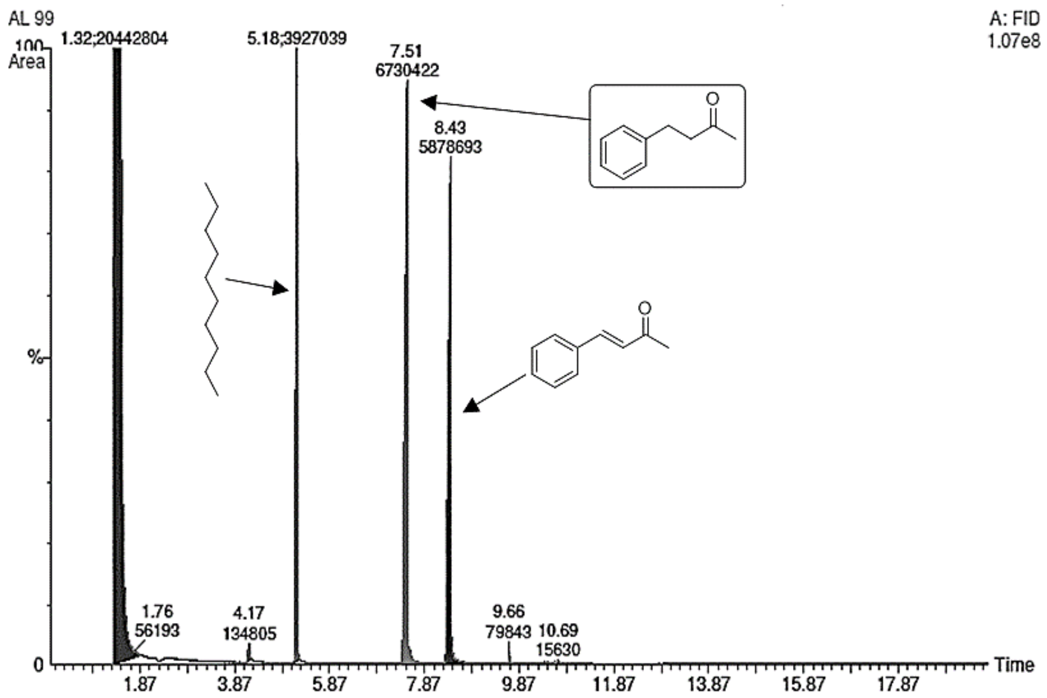

Figure S1. Typical chromatogram for the hydrogenation reaction of *(E)*-4-phenylbut-3-en-2-one (**1**), for the third use of the catalytic system **Pd NPs/N00Cl-100**.

In order to determine the results, *i.e.* substrate conversions and yield products, in the case of the hydrogenation of *(E)*-4-phenylbut-3-en-2-one to 4-phenylbutan-2-one, both compounds were previously calibrated in GC using *n*-decane as standard. We gather the used experimental calibrating equations:

$$\frac{(E)-4-phenylbut-3-en-2-one\ mass}{n-decane\ mass} = 1.5533 \cdot \frac{(E)-4-phenylbut-3-en-2-one\ area}{n-decane\ area}$$ **Equation S1**

$$\frac{4-phenylbutan-2-one\ mass}{n-decane\ mass} = k \cdot \frac{4-phenylbutan-2-one\ area}{n-decane\ area}$$ **Equation S2**

From calibration equations S1 and S2, we determined the reaction results using the next equations:

$$Conv.(\%) = \left(1 - 1.64408 \cdot \frac{4-phenylbut-3-en-2-one\ area\ .\ n-decane\ mg}{n-decane\ area\ .\ 4-phenylbut-3-en-2-one\ mg}\right) \cdot 100$$ **Equation S3**

$$Yield\ (\%) = \left(1.335817 \cdot \frac{4-phenylbutan-2-one\ area\ .\ n-decane\ mg}{n-decane\ area\ .\ 4-phenylbut-3-en-2-one\ mg}\right) \cdot 100$$ **Equation S4**

These results were also determined by ^{1}H NMR for all the reactions using *n*-decane as standard, in order to confirm the obtained values.

In the case of the hydrogenation of the rest of substrates, the results were determined by ^{1}H NMR, using 1,4-dioxane as standard.

In the case of the hydrodehalogenation reactions, the identity of all the substrates and compounds was confirmed according to their mass spectra. The results were obtained by GC, previous GC calibration using *n*-decane as standard, as well as by NMR using 1,4-dioxane as standard. We gather here the experimental calibrating equation:

$$\frac{product\ mass}{n-decane\ mass} = k \cdot \frac{product\ area}{n-decane\ area} + b$$ **Equation S5**

where ***k*** = 2.2401 (for 1,4-bromoclorobenzene); 1.7725 (for bromobenzene); 1.1056 (for chlorobenzene); 0.5488 (for benzene); 2.8579 (for trichlorobenzene); and with ***b*** = 0.1783 (for 1,4-bromoclorobenzene); 0.1102 (for bromobenzene); 0.0856 (for chlorobenzene); 0.4595 (for benzene); 0.1083 (for trichlorobenzene); being respectively r^2 = 0.9999; 0.9995; 0.9967; 0.9985 and 0.9992; etc.

From calibration equation S5, the reaction results were determined using the next equations:

$$Conversion\ (\%) = \left(1 - k \cdot \frac{substrate\ area\ .\ n-decane\ mass}{n-decane\ area\ .\ substrate\ mass}\right) \cdot 100$$ **Equation S6**

$$Yield\ (\%) = \left(\frac{n-decane\ mass\ .\ \left(k \cdot \frac{product\ area}{n-decane\ area} + b\right)}{mmol\ substrate\ .\ Mm\ product}\right) \cdot 100$$ **Equation S7**

3. - Characterization of used glycerol-based solvents

Glycerol-based solvents were purified by vacuum distillation prior to use, and characterized by ^{1}H NMR, ^{13}C NMR, ^{19}F NMR, HRMS, IR and GC-MS. NMR spectra (in DMSO-d$_6$, δ ppm, *J* in Hz) were obtained using a Bruker Avance 400 MHz spectrometer. HRMS was carried out in a Bruker MicroTof-Q spectrometer with electrospray ionization in methanol. IR spectra were acquired in a Nicolet 5700 FT-IR spectrometer in the range of 4000–400 cm^{-1}. GC-MS analyses were done in a HP 6890 Series II chromatograph equipped with a ZB-5HT Inferno column and coupled to an Agilent 5973 inert mass detector with electronic impact system. Solvents boiling points were determined with the onset temperature of differential scanning calorimetric analysis in a TA Instruments DSC-Q20, calibrated with indium, and using micropore aluminium pans at atmospheric pressure. Solvents water content was controlled using a Schott Karl Fischer titrator. Solvents viscosity was measured in triplicate using different Ubbelohde viscometers with suspended ball level with uncertainties lower than 0.2% (0.5% in the case of DES).

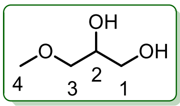

3-Methoxy-1,2-propanediol [100]: colorless liquid, b.p. = 222 ºC. **^{1}H NMR** (400 MHz, DMSO-d$_6$, 25 ºC): δ 4.61 (d, 1H, *J* = 5.1 Hz, OH^{2}), 4.46 (t, 1H, *J* = 5.7 Hz, OH^{1}), 3.56 (sext, 1H, *J* = 5.2 Hz, H^{2}), 3.26-3.36 (m, 3H, H^{1}, H^{3a}), 3.23 (s, 3H, H^{4}), 3.21 (dd, 1H, J_{gem} = 9.8 Hz, *J* = 6.0 Hz, H^{3b}). **^{13}C NMR** (100 MHz, DMSO-d$_6$, 25 ºC): δ 74.2 (CH$_2$, C^{3}), 70.3 (CH, C^{2}), 62.9 (CH$_2$, C^{1}), 58.3 (CH$_3$, C^{4}). **HRMS** (ESI$^+$, M+Na$^+$): *m/z* calc. = 129.0522, *m/z* found = 129.0527. **IR** (ATR): ν_{max} 3376, 2926, 2884, 2822, 1457, 1327, 1195, 1127, 1109, 1042 cm^{-1}. **GC-MS** (EI) *m/z*: 88, 75, 61, 45 (100%), 44, 43, 31.

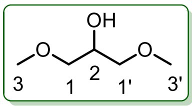

1,3-Dimethoxy-2-propanol [101]: colorless liquid, b.p. = 170 ºC. **^{1}H NMR** (400 MHz, DMSO-d$_6$, 25 ºC): δ 4.79 (d, 1H, *J* = 5.2 Hz, OH^{2}), 3.70 (sext, 1H, *J* = 5.1 Hz, H^{2}), 3.28 (dd, 2H, J_{gem} = 9.8 Hz, *J* = 5.0 Hz, H^{1a}, H$^{1a'}$), 3.23 (dd, 2H, J_{gem} = 9.9 Hz, *J* = 5.6 Hz, H^{1b}, H$^{1b'}$), 3.24 (s, 6H, H^{3}, H$^{3'}$). **^{13}C NMR** (100 MHz, DMSO-d$_6$, 25 ºC): δ 74.2 (CH$_2$, C$^{1/1'}$), 68.2 (CH, C^{2}), 58.4 (CH$_3$, C$^{3/3'}$). **HRMS** (ESI$^+$, M+Na$^+$): *m/z* calc. = 143.0679, *m/z* found = 143.0681. **IR** (ATR): ν_{max} 3444, 2982, 2926, 2887, 2820, 1454, 1330, 1195, 1106, 1027 cm^{-1}. **GC-MS** (EI) *m/z*: 75, 71, 45 (100%), 43, 31.

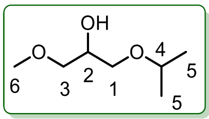

1-Isopropoxy-3-methoxy-2-propanol [103i]: colorless liquid, b.p. = 184 ºC. **^{1}H NMR** (400 MHz, DMSO-d$_6$, 25 ºC): δ 4.70 (d, 1H, *J* = 5.1 Hz, OH^{2}), 3.64 (sext, 1H, *J* = 5.5 Hz, H^{2}), 3.51 (sept, 1H, *J* = 5.9 Hz, H^{4}), 3.20-3.32 (m, 4H, H^{1}, H^{3}), 3.24 (s, 3H, H^{6}), 1.06 (d, 6H, *J* = 6.1 Hz, H^{5}). **^{13}C NMR** (100 MHz, DMSO-d$_6$, 25 ºC): δ 74.4 (CH$_2$, C^{3}), 71.0 (CH, C^{4}), 69.5 (CH$_2$, C^{1}), 68.7 (CH, C^{2}), 58.4 (CH$_3$, C^{6}), 22.0 (CH$_3$, C^{5}). **HRMS** (ESI$^+$, M+Na$^+$): *m/z* calc. = 171.0992, *m/z* found = 171.0994. **IR** (ATR): ν_{max} 3432, 2973, 2929, 2876, 1457, 1368, 1336, 1124, 1079 cm^{-1}. **GC-MS** (EI) *m/z:* 103, 89, 75, 73, 61, 59, 57, 45 (100%), 43, 41, 31.

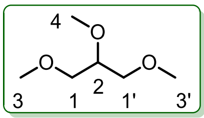

1,2,3-Trimethoxypropane [111]: colorless liquid, b.p. = 150 ºC. **^{1}H NMR** (400 MHz, DMSO-d$_6$, 25 ºC): δ 3.39-3.43 (m, 1H, H^{2}), 3.32-3.39 (m, 4H, H^{1}, H$^{1'}$), 3.31 (s, 3H, H^{4}), 3.25 (s, 6H, H^{3}, H$^{3'}$). **^{13}C NMR** (100 MHz, DMSO-d$_6$, 25 ºC): δ 78.5 (CH, C^{2}), 71.8 (CH$_2$, C$^{1/1'}$), 58.4 (CH$_3$, C$^{3/3'}$), 57.0 (CH$_3$, C^{4}). **HRMS** (ESI$^+$, M+Na$^+$): *m/z* calc. = 157.0835, *m/z* found = 157.0857. **IR** (ATR): ν_{max} 2982, 2931, 2881, 2817, 1451, 1359, 1336, 1191, 1086 cm^{-1}. **GC-MS** (EI) *m/z:* 102, 89, 71, 59, 45 (100%), 43, 31.

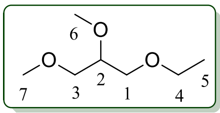

1-Ethoxy-2,3-dimethoxypropane [112]: colorless liquid, b.p. = 158 ºC. **^{1}H NMR** (400 MHz, DMSO-d$_6$, 25 ºC): δ 3.40-3.45 (m, 2H, H^{1}), 3.32-3.40 (m, 5H, H^{2}, H^{3}, H^{4}), 3.31 (s, 3H, H^{6}), 3.24 (s, 3H, H^{7}), 1.09 (t, 3H, *J* = 7.0 Hz, H^{5}). **^{13}C NMR** (100 MHz, DMSO-d$_6$, 25 ºC): δ 78.6 (CH, C^{2}), 71.8 (CH$_2$, C^{3}), 69.6 (CH$_2$, C^{4}), 65.8 (CH$_2$, C^{1}), 58.4 (CH$_3$, C^{7}), 57.0 (CH$_3$, C^{6}), 15.0 (CH$_3$, C^{5}). **HRMS** (ESI$^+$, M+Na$^+$): *m/z* calc. = 171.0992,

m/z found = 171.0993. **IR** (ATR): ν_{max} 2977, 2926, 2855, 1460, 1377, 1333, 1195, 1112 cm^{-1}. **GC-MS** (EI) *m/z:* 116, 103, 89, 75, 72, 59 (100%), 57, 45, 43, 31.

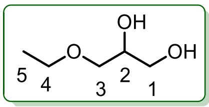

3-Ethoxy-1,2-propanediol [200]: colorless liquid, b.p. = 221 ºC. **^{1}H NMR** (400 MHz, DMSO-d_6, 25 ºC): δ 4.59 (d, 1H, *J* = 5.1 Hz, OH^2), 4.45 (t, 1H, *J* = 5.7 Hz, OH^1), 3.55 (sext, 1H, *J* = 5.3 Hz, H^2), 3.42 (q, 2H, *J* = 7.0 Hz, H^4), 3.21-3.37 (m, 4H, H^1, H^3), 1.10 (t, 3H, *J* = 7.0 Hz, H^5). **^{13}C NMR** (100 MHz, DMSO-d_6, 25 ºC): δ 72.3 (CH_2, C^3), 70.8 (CH, C^2), 66.0 (CH_2, C^4), 63.4 (CH_2, C^1), 15.4 (CH_3, C^5). **HRMS** (ESI^+, $M+Na^+$): *m/z* calc. = 143.0679, *m/z* found = 143.0680. **IR** (ATR): ν_{max} 3382, 2976, 2929, 2870, 1445, 1380, 1174, 1112, 1042 cm^{-1}. **GC-MS** (EI) *m/z*: 89, 61, 59, 45, 44, 43, 31 (100%).

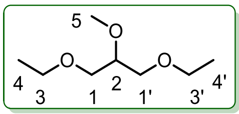

1,3-Diethoxy-2-methoxypropane [212]: colorless liquid, b.p. = 173 ºC. **^{1}H NMR** (400 MHz, DMSO-d_6, 25 ºC): δ 3.42 (q, 4H, *J* = 6.9 Hz, H^3, $H^{3'}$), 3.33-3.41 (m, 5H, H^1, $H^{1'}$, H^2), 3.31 (s, 3H, H^5), 1.10 (t, 6H, *J* = 7.0 Hz, H^4, $H^{4'}$). **^{13}C NMR** (100 MHz, DMSO-d_6, 25 ºC): δ 78.9 (CH, C^2), 69.7 (CH_2, $C^{1/1'}$), 65.8 (CH_2, $C^{3/3'}$), 57.0 (CH_3, C^5), 15.1 (CH_3, $C^{4/4'}$). **HRMS** (ESI^+, $M+Na^+$): *m/z* calc. = 185.1148, *m/z* found = 185.1152. **IR** (ATR): ν_{max} 2976, 2931, 2864, 1445, 1380, 1333, 1197, 1112 cm^{-1}. **GC-MS** (EI) *m/z:* 130, 103, 86, 75 (100%), 58, 45, 43, 31.

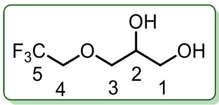

3-(2,2,2-Trifluoroethoxy)-1,2-propanediol [3F00]: colorless liquid, b.p. = 213 ºC. **^{1}H NMR** (400 MHz, DMSO-d_6, 25 ºC): δ 4.80 (d, 1H, *J* = 5.2 Hz, OH^2), 4.57 (t, 1H, *J* = 5.6 Hz, OH^1), 4.05 (q, 2H, *J* = 9.4 Hz, H^4), 3.57-3.64 (m, 2H, H^2, H^{3a}), 3.45-3.52 (m, 1H, H^{3b}), 3.28-3.36 (m, 2H, H^1). **^{13}C NMR** (100 MHz, DMSO-d_6, 25 ºC): δ 124.6 (q, CF_3, *J* = 279.6 Hz, C^5), 73.9 (CH_2, C^3), 70.5 (CH, C^2), 67.7 (q, CH_2, *J* = 32.6 Hz, C^4), 62.7 (CH_2, C^1). **^{19}F NMR** (400 MHz, DMSO-d_6, 25 ºC): δ –73.0 (t, CF_3, *J* = 9.4 Hz). **HRMS** (ESI^+, $M+Na^+$): *m/z* calc. = 197.0396, *m/z* found = 197.0400. **IR** (ATR): ν_{max} 3367, 2940, 2887, 1460, 1416, 1277, 1168, 1112, 1053 cm^{-1}. **GC-MS** (EI) *m/z*: 143, 113, 83, 69, 61, 44, 43, 31 (100%).

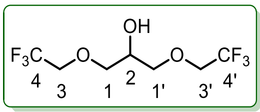

1,3-Bis(2,2,2-trifluoroethoxy)-2-propanol [3F03F]: colorless liquid, b.p. = 197 ºC. **^{1}H NMR** (400 MHz, DMSO-d_6, 25 ºC): δ 5.13 (d, 1H, *J* = 5.3 Hz, OH^2), 4.07 (q, 4H, *J* = 9.4 Hz, H^3, $H^{3'}$), 3.78 (sext, 1H, *J* = 5.3 Hz, H^2), 3.58 (dd, 2H, J_{gem} = 10.2 Hz, *J* = 4.8 Hz, H^{1a}, $H^{1a'}$), 3.53 (dd, 2H, J_{gem} = 10.2 Hz, *J* = 5,8 Hz, H^{1b}, $H^{1b'}$). **^{13}C NMR** (100 MHz, DMSO-d_6, 25 ºC): δ 124.5 (q, CF_3, *J* = 279.6 Hz, $C^{4/4'}$), 73.3 (CH_2, $C^{1/1'}$), 68.2 (CH, C^2), 67.6 (q, CH_2, *J* = 32.6 Hz, $C^{3/3'}$). **^{19}F NMR** (400 MHz, DMSO-d_6, 25 ºC): δ –72.8 (t, CF_3, *J* = 9.4 Hz). **HRMS** (ESI^+, $M+Na^+$): *m/z* calc. = 279.0426, *m/z* found = 279.0426. **IR** (ATR): ν_{max} 3482, 2937, 2880, 1460, 1445, 1310, 1277, 1165 cm^{-1}. **GC-MS** (EI) *m/z*: 143 (100%), 123, 113, 95, 83, 75, 57, 43, 31.

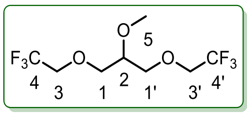

2-Methoxy-1,3-bis(2,2,2-trifluoroethoxy)propane [3F13F]: Colorless liquid, b.p. = 178 ºC. **^{1}H NMR** (400 MHz, DMSO-d_6, 25 ºC): δ 4.06 (q, 4H, *J* = 9.3 Hz, H^3, $H^{3'}$), 3.69 (dd, 2H, J_{gem} = 10.6 Hz, *J* = 4.9 Hz, H^{1a}, $H^{1a'}$), 3.63 (dd, 2H, J_{gem} = 10.6 Hz, *J* = 5.4 Hz, H^{1b}, $H^{1b'}$), 3.51 (quint, 1H, *J* = 5.2 Hz, H^2), 3.35 (s, 3H, H^5). **^{13}C NMR** (100 MHz, DMSO-d_6, 25 ºC): δ 124.4 (q, CF_3, *J* = 279.4 Hz, $C^{4/4'}$), 78.4 (CH, C^2), 71.0 (CH_2, $C^{1/1'}$), 67.6 (q, CH_2, *J* = 32.8 Hz, $C^{3/3'}$), 57.1 (CH_3, C^5). **^{19}F NMR** (400 MHz, DMSO-d_6, 25 ºC): δ –73.3 (t, CF_3, *J* = 9.3 Hz). **HRMS** (ESI^+, $M+Na^+$): *m/z* calc. = 293.0583, *m/z* found = 293.0571. **IR** (ATR): ν_{max} 2940, 2890, 2840, 1460, 1277, 1150 cm^{-1}. **GC-MS** (EI) *m/z*: 157 (100%), 139, 127, 113, 83, 69, 45.

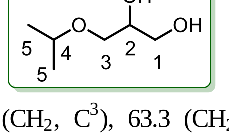

3-Isopropoxy-1,2-propanediol [3i00]: colorless liquid, b.p. = 202 ºC. **^{1}H NMR** (400 MHz, DMSO-d_6, 25 ºC): δ 4.52 (d, 1H, *J* = 5.0 Hz, OH^2), 4.42 (t, 1H, *J* = 5.7 Hz, OH^1), 3.46-3.56 (m, 2H, H^2, H^4), 3.22-3.38 (m, 4H, H^1, H^3), 1.07 (d, 6H, *J* = 6.1 Hz, H^5). **^{13}C NMR** (100 MHz, DMSO-d_6, 25 ºC): δ 71.0 (CH, C^2, C^4), 69.7 (CH_2, C^3), 63.3 (CH_2, C^1), 22.1 (CH_3, C^5). **HRMS** (ESI^+, $M+Na^+$): *m/z* calc. = 157.0835, *m/z* found = 157.0839. **IR** (ATR): ν_{max} 3382, 2973, 2931, 2873, 1469, 1368, 1177, 1127, 1039 cm^{-1}. **GC-MS** (EI) *m/z*: 73, 61, 57, 44, 43 (100%), 39, 31.

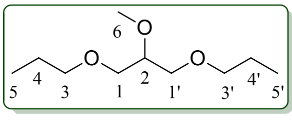

1,3-Dipropoxy-2-methoxypropane [313]: colorless liquid, b.p. = 180 ºC. **^{1}H NMR** (400 MHz, DMSO-d_6, 25 ºC): δ 3.35-3.43 (m, 5H, H^1, $H^{1'}$, H^2),

3.33 (t, 4H, *J* = 6.6 Hz, H^{3}, $H^{3'}$), 3.32 (s, 3H, H^{6}), 1.49 (sext, 4H, *J* = 7.3 Hz, H^{4}, $H^{4'}$), 0.85 (t, 6H, *J* = 7.4 Hz, H^{5}, $H^{5'}$). **^{13}C NMR** (100 MHz, DMSO-d_6, 25 °C): δ 78.8 (CH, C_2), 72.1 (CH_2, $C^{3/3'}$), 69.8 (CH_2, $C^{1/1'}$), 57.0 (CH_3, C^{6}), 22.4 (CH_2, $C^{4/4'}$), 10.5 (CH_3, $C^{5/5'}$). **HRMS** (ESI$^+$, M+Na$^+$): *m/z* calc. = 213.1461, *m/z* found = 213.1468. **IR** (ATR): ν_{max} 2961, 2934, 2864, 1463, 1380, 1333, 1198, 1112 cm^{-1}. **GC-MS** (EI) *m/z:* 100, 89, 75, 58, 43 (100%), 41.

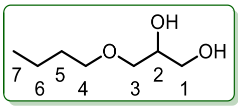

3-Butoxy-1,2-propanediol [400]: colorless liquid, b.p. = 250 °C. **^{1}H NMR** (400 MHz, DMSO-d_6, 25 °C): δ 4.54 (d, 1H, *J* = 5.1 Hz, OH^{2}), 4.41 (t, 1H, *J* = 5.7 Hz, OH^{1}), 3.53 (sext, 1H, *J* = 5.2 Hz, H^{2}), 3.34 (t, 2H, *J* = 6.7 Hz, H^{4}), 3.21-3.32 (m, 4H, H^{1}, H^{3}), 1.44 (quint, 2H, *J* = 7.6 Hz, H^{5}), 1.29 (sext, 2H, *J* = 7.3 Hz, H^{6}), 0.85 (t, 3H, *J* = 7.4 Hz, H^{7}). **^{13}C NMR** (100 MHz, DMSO-d_6, 25 °C): δ 72.3 (CH_2, C^{3}), 70.6 (CH, C^{2}), 70.2 (CH_2, C^{4}), 63.2 (CH_2, C^{1}), 31.4 (CH_2, C^{5}), 18.9 (CH_2, C^{6}), 13.8 (CH_3, C^{7}). **HRMS** (ESI$^+$, M+Na$^+$): *m/z* calc. = 171.0992, *m/z* found = 171.0996. **IR** (ATR): ν_{max} 3388, 2958, 2931, 2867, 1463, 1377, 1115, 1048 cm^{-1}. **GC-MS** (EI) *m/z*: 117, 87, 61, 57 (100%), 44, 43, 41, 31.

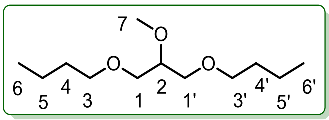

1,3-Dibutoxy-2-methoxypropane [414]: colorless liquid, b.p. = 244 °C. **^{1}H NMR** (400 MHz, DMSO-d_6, 25 °C): δ 3.32-3.47 (m, 5H, H^{1}, $H^{1'}$, H^{2}), 3.37 (t, 4H, *J* = 6.6 Hz, H^{3}, $H^{3'}$), 3.31 (s, 3H, H^{7}), 1.47 (quint, 4H, *J* = 6.2 Hz, H^{4}, $H^{4'}$), 1.31 (sext, 4H, *J* = 7.2 Hz, H^{5}, $H^{5'}$), 0.87 (t, 6H, *J* = 7.3 Hz, H^{6}, $H^{6'}$). **^{13}C NMR** (100 MHz, DMSO-d_6, 25 °C): δ 78.8 (CH, C^{2}), 70.2 (CH_2, $C^{3/3'}$), 69.9 (CH_2, $C^{1/1'}$), 57.0 (CH_3, C^{7}), 31.3 (CH_2, $C^{4/4'}$), 18.8 (CH_2, $C^{5/5'}$), 13.7 (CH_3, $C^{6/6'}$). **HRMS** (ESI$^+$, M+Na$^+$): *m/z* calc. = 241.1774, *m/z* found = 241.1776. **IR** (ATR): ν_{max} 2958, 2931, 2864, 1466, 1377, 1198, 1112 cm^{-1}. **GC-MS** (EI) *m/z:* 130, 114, 87, 75, 59, 57 (100%), 41, 31.

GLYCEROL-DERIVED EUTECTIC SOLVENTS

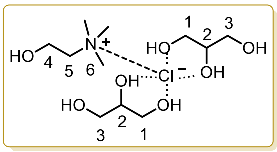

1:2 Choline chloride/glycerol [ChCl-000]: colorless liquid. **^{1}H NMR** (400 MHz, DMSO-d_6, 25 °C): δ 5.50 (t, 1H, *J* = 5.1 Hz, OH^{4}), 4.55 (d, 2H, *J* = 4.7 Hz, OH^{2}), 4.48 (t, 4H, *J* = 5.7 Hz, $OH^{1/3}$), 3.77-3.85 (m, 2H, H^{4}), 3.38-3.45 (m, 4H, H^{2}, H^{5}), 3.35 (dt, 4H, J_{gem} = 11.1 Hz, *J* = 4.1 Hz, $H^{1a/3a}$), 3.27 (dt, 4H, J_{gem} = 11.0 Hz, *J* = 5.6 Hz, $H^{1b/3b}$), 3.13 (s, 9H, H^{6}). **^{13}C NMR** (100 MHz, DMSO-d_6, 25 °C): δ 72.6 (CH, C^{2}), 67.0 (CH_2, C^{5}), 63.1 (CH_2, $C^{1/3}$), 55.1 (CH_2, C^{4}), 53.2 (CH_3, C^{6}). **IR** (ATR): ν_{max} 3326, 2929, 2878, 1477, 1416, 1109, 1048 cm^{-1}.

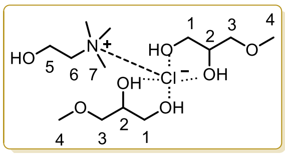

1:2 Choline chloride/3-methoxy-1,2-propanediol [ChCl-100]: colorless liquid. **^{1}H NMR** (400 MHz, DMSO-d_6, 25 °C): δ 5.55 (t, 1H, *J* = 5.1 Hz, OH^{5}), 4.70 (d, 2H, *J* = 5.1 Hz, OH^{2}), 4.57 (t, 2H, *J* = 5.7 Hz, OH^{1}), 3.78-3.85 (m, 2H, H^{5}), 3.55 (sext, 2H, *J* = 5.2 Hz, H^{2}), 3.41-3.44 (m, 2H, H^{6}), 3.23 (s, 6H, H^{4}), 3.25-3.34 (m, 6H, H^{1}, H^{3a}), 3.15-3.24 (m, 2H, H^{3b}), 3.13 (s, 9H, H^{7}). **^{13}C NMR** (100 MHz, DMSO-d_6, 25 °C): δ 74.2 (CH_2, C^{3}), 70.5 (CH, C^{2}), 67.0 (CH_2, C^{6}), 63.2 (CH_2, C^{1}), 58.4 (CH_3, C^{4}), 55.1 (CH_2, C^{5}), 53.2 (CH_3, C^{7}). **IR** (ATR): ν_{max} 3326, 2930, 2929, 2884, 2822, 1477, 1418, 1195, 1127, 1089, 1045 cm^{-1}.

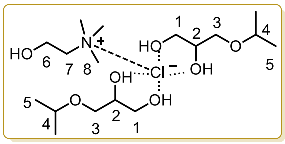

1:2 Choline chloride/3-isopropoxy-1,2-propanediol [ChCl-3i00]: colorless liquid. **^{1}H NMR** (400 MHz, DMSO-d_6, 25 °C): δ 5.53-5.57 (m, 1H, OH^{6}), 4.58-4.63 (m, 2H, OH^{2}), 4.48-4.54 (m, 2H, OH^{1}), 3.78-3.85 (m, 2H, H^{6}), 3.46-3.56 (m, 4H, H^{2}, H^{4}), 3.41-3.45 (m, 2H, H^{7}), 3.20-3.39 (m, 8H, H^{1}, H^{3}), 3.14 (s, 9H, H^{8}), 1.06 (d, 12H, *J* = 6.1 Hz, H^{5}). **^{13}C NMR** (100 MHz, DMSO-d_6, 25 °C): δ 71.0 (CH, C^{2}, C^{4}), 69.6 (CH_2, C^{3}), 66.9 (CH_2, C^{7}), 63.3 (CH_2, C^{1}), 55.1 (CH_2, C^{6}), 53.1 (CH_3, C^{8}), 22.1 (CH_3, C^{5}). **IR** (ATR): ν_{max} 3334, 2970, 2926, 2872, 1474, 1380, 1336, 1133, 1074 cm^{-1}.

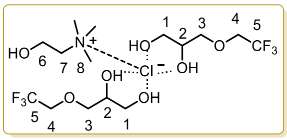

1:2 Choline chloride/3-(trifluoroethoxy)-1,2-propanediol [ChCl-3F00]: colorless liquid. **^{1}H NMR** (400 MHz, DMSO-d_6, 25 °C): δ 5.56 (t, 1H, *J* = 5.1 Hz, OH^{6}), 4.91 (d, 2H, *J* = 4.9 Hz, OH^{2}), 4.69 (t, 2H, *J* = 5.7 Hz, OH^{1}), 4.04 (q, 4H, *J* = 9.4 Hz, H^{4}), 3.78-3.84 (m, 2H, H^{6}), 3.46-3.66 (m, 6H, H^{2},

H^3), 3.42-3.46 (m, 2H, H^7), 3.31 (dd, 4H, J_{gem} = 11.0 Hz, J = 5.9 Hz, H^1), 3.15 (s, 9H, H^8). **^{13}C NMR** (100 MHz, DMSO-d_6, 25 ºC): δ 124.6 (q, CF_3, J = 277.0 Hz, C^5), 73.8 (CH_2, C^3), 70.6 (CH, C^2), 67.7 (q, CH_2, J = 32.0 Hz, C^4), 67.0 (CH_2, C^7), 62.7 (CH_2, C^1), 55.1 (CH_2, C^6), 53.2 (CH_3, C^8). **IR** (ATR): ν_{max} 3320, 2931, 2884, 1480, 1421, 1277, 1165, 1050 cm^{-1}.

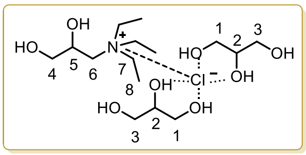

1:2 *N,N,N*-Triethyl-2,3-dihydroxypropan-1-aminium chloride/glycerol [N00Cl-000]: colorless liquid. **^{1}H NMR** (400 MHz, DMSO-d_6, 25 ºC): δ 5.59 (d, 1H, J = 5.9 Hz, OH^5), 5.20 (t, 1H, J = 5.7 Hz, OH^4), 4.55 (d, 2H, J = 4.7 Hz, OH^2), 4.48 (t, 4H, J = 5.7 Hz, $OH^{1/3}$), 3.92-4.03 (m, 1H, H^5), 3.16-3.47 (m, 20H, H^1, H^2, H^3, H^4, H^6, H^7), 1.18 (t, 9H, J = 7.1 Hz, H^8). **^{13}C NMR** (100 MHz, DMSO-d_6, 25 ºC): δ 73.0 (CH, C^2), 65.9 (CH, C^5), 64.1 (CH_2, C^4), 63.5 (CH_2, $C^{1/3}$), 59.9 (CH_2, C^6), 53.4 (CH_2, C^7), 7.7 (CH_3, C^8). **IR** (ATR): 3329, 2931, 2876, 1463, 1398, 1112, 1042 cm^{-1}.

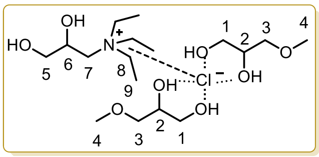

1:2 *N,N,N*-Triethyl-2,3-dihydroxypropan-1-aminium chloride/3-methoxy-1,2-propanediol [N00Cl-100]: colorless liquid. **^{1}H NMR** (400 MHz, DMSO-d_6, 25 ºC): δ 5.65 (d, 1H, J = 5.8 Hz, OH^6), 5.24 (t, 1H, J = 5.3 Hz, OH^5), 4.70 (d, 2H, J = 4.9 Hz, OH^2), 4.57 (t, 2H, J = 5.6 Hz, OH^1), 3.92-4.01 (m, 1H, H^6), 3.55 (sext, 2H, J = 5.1 Hz, H^2), 3.22 (s, 6H, H^4), 3.15-3.47 (m, 18H, H^1, H^3, H^5, H^7, H^8), 1.18 (t, 9H, J = 7.1 Hz, H^9). **^{13}C NMR** (100 MHz, DMSO-d_6, 25 ºC): δ 74.3 (CH_2, C^3), 70.4 (CH, C^2), 65.5 (CH, C^6), 63.7 (CH_2, C^5), 63.0 (CH_2, C^1), 59.5 (CH_2, C^7), 58.4 (CH_3, C^4), 52.9 (CH_2, C^8), 7.3 (CH_3, C^9). **IR** (ATR): ν_{max} 3320, 2984, 2929, 2884, 2826, 1457, 1398, 1192, 1127, 1095, 1042 cm^{-1}.

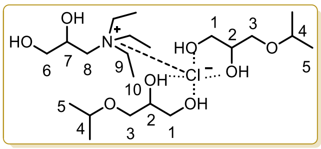

1:2 *N,N,N*-Triethyl-2,3-dihydroxypropan-1-aminium chloride/3-isopropoxy-1,2-propanediol [N00Cl-3i00]: colorless liquid. **^{1}H NMR** (400 MHz, DMSO-d_6, 25 ºC): δ 5.48 (d, 1H, J = 5.5 Hz, OH^7), 5.15 (dd, 1H, J = 6.1 Hz, J = 5.1 Hz, OH^6), 4.55 (d, 2H, J = 5.0 Hz, OH^2), 4.45 (t, 2H, J = 5.7 Hz, OH^1), 3.92-4.00 (m, 1H, H^7), 3.47-3.55 (m, 4H, H^2, H^4), 3.16-3.46 (m, 18H, H^1, H^3, H^6, H^8, H^9), 1.18 (t, 9H, J = 7.1 Hz, H^{10}), 1.07 (d, 12H, J = 6.1 Hz, H^5). **^{13}C NMR** (100 MHz, DMSO-d_6, 25 ºC): δ 71.0 (CH, C^2, C^4), 69.6 (CH_2, C^3), 65.6 (CH, C^7), 63.6 (CH_2, C^6), 63.3 (CH_2, C^1), 59.4 (CH_2, C^8), 52.8 (CH_2, C^9), 22.1 (CH_3, C^5), 7.2 (CH_3, C^{10}). **IR** (ATR): ν_{max} 3323, 2970, 2929, 2870, 1469, 1368, 1127, 1047 cm^{-1}.

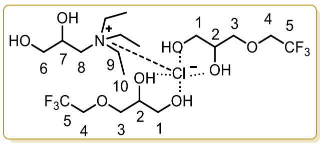

1:2 *N,N,N*-Triethyl-2,3-dihydroxypropan-1-aminium chloride/3-(2,2,2-trifluoroethoxy)-1,2-propanediol [N00Cl-3F00]: colorless liquid. **^{1}H NMR** (400 MHz, DMSO-d_6, 25 ºC): δ 5.65 (d, 1H, J = 5.1 Hz, OH^7), 5.24 (t, 1H, J = 5.3 Hz, OH^6), 4.88 (d, 2H, J = 4.7 Hz, OH^2), 4.67 (t, 2H, J = 5.6 Hz, OH^1), 4.04 (q, 4H, J = 9.6 Hz, H^4), 3.92-4.00 (m, 1H, H^7), 3.55-3.63 (m, 4H, H^2, H^{3a}), 3.49 (dd, 2H, J_{gem} = 10.4 Hz, J = 5.0 Hz, H^{3b}), 3.16-3.45 (m, 10H, H^6, H^8, H^9), 3.32 (dd, 4H, J = 5.9 Hz, J = 5.7 Hz, H^1), 1.18 (t, 9H, J = 7.1 Hz, H^{10}). **^{13}C NMR** (100 MHz, DMSO-d_6, 25 ºC): δ 124.6 (q, CF_3, J = 279.6 Hz, C^5), 73.9 (CH_2, C^3), 70.6 (CH, C^2), 67.6 (q, CH_2, J = 32.5 Hz, C^4), 65.6 (CH, C^7), 63.7 (CH_2, C^6), 62.7 (CH_2, C^1), 59.5 (CH_2, C^8), 52.9 (CH_2, C^9), 7.3 (CH_3, C^{10}). **IR** (ATR): ν_{max} 3320, 2937, 2881, 1460, 1398, 1277, 1168 cm^{-1}.

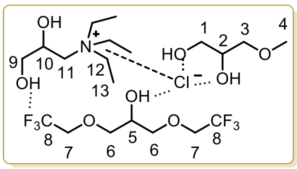

1:1:1 *N,N,N*-Triethyl-2,3-dihydroxypropan-1-aminium chloride/3-methoxy-1,2-propanediol/1,3-bis(2,2,2-trifluoroethoxy)-2-propanol [N00Cl-100-3F03F]: colorless liquid. **^{1}H NMR** (400 MHz, DMSO-d_6, 25 ºC): δ 5.66 (d, 1H, J = 5.7 Hz, OH^{10}), 5.23 (t, 1H, J = 5.6 Hz, OH^9), 5.20 (d, 1H, J = 5.2 Hz, OH^5), 4.68 (d, 1H, J = 5.0 Hz, OH^2), 4.55 (t, 1H, J = 5.7 Hz, OH^1), 4.06 (q, 4H, J = 9.4 Hz, H^7, $H^{7'}$), 3.94-4.01 (m, 1H, H^{10}), 3.77 (sext, 1H, J = 5.2 Hz, H^5), 3.51-3.60 (m, 5H, H^2, H^6, $H^{6'}$), 3.23 (s, 3H, H^4), 3.15-3.48 (m, 14H, H^1, H^3, H^9, H^{11}, H^{12}), 1.19 (t, 9H, J = 7.1 Hz, H^{13}). **^{13}C NMR** (100 MHz, DMSO-d_6, 25 ºC): δ 124.5 (q, CF_3, J = 278.0 Hz, $C^{8/8'}$), 74.3 (CH_2, C^3), 73.3 (CH_2, $C^{6/6'}$), 70.4 (CH, C^2), 68.2 (CH, C^5), 67.6 (q, CH_2, J = 32.0 Hz, $C^{7/7'}$), 65.5 (CH, C^{10}), 63.7 (CH_2, C^9), 63.1 (CH_2, C^1), 59.5 (CH_2, C^{11}), 58.3 (CH_3, C^4), 52.9 (CH_2, C^{12}), 7.2 (CH_3, C^{13}). **^{19}F NMR** (400 MHz, DMSO-d_6, 25 ºC): δ –73.0 (t, CF_3, J = 9.4 Hz). **IR** (ATR): ν_{max} 3302, 2987, 2934, 2881, 1463, 1398, 1277, 1162 cm^{-1}.

4. - Characterization of catalytic systems

All the synthesized **Pd NPs/glycerol-based solvent** catalytic systems were characterized by Transmission Electron Microscopy (TEM). TEM micrographs were obtained using a JEOL JEM 1400 microscope running at 120 kV (Université de Toulouse III-Paul Sabatier & LHFA-CNRS). Pd NPs size distribution diagrams and mean diameters were obtained from TEM images, applying Image-J software associated to a Microsoft Excel macro.

4.1. – TEM analyses

We gather next two transmission electron micrographs (selected from a total of up 10 images acquired) and the size distribution diagrams (average results) for the Pd NPs immobilized in each of the glycerol-based solvents.

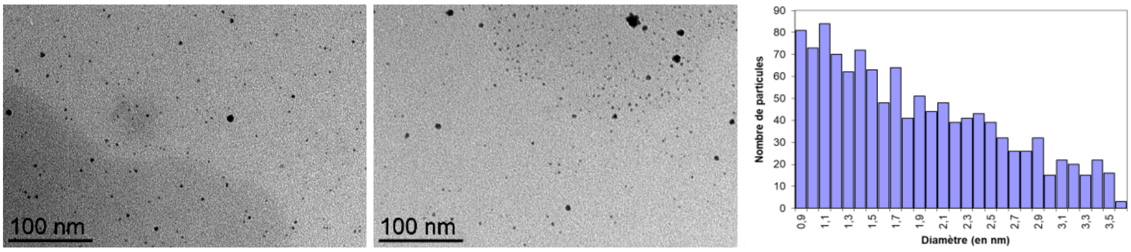

Figure S2. TEM micrographs and size distribution (nm) of the PVP-Pd NPs immobilized in glycerol (**000**).
Medium size: 1.5 ± 0.8 nm (for 1664 particles).

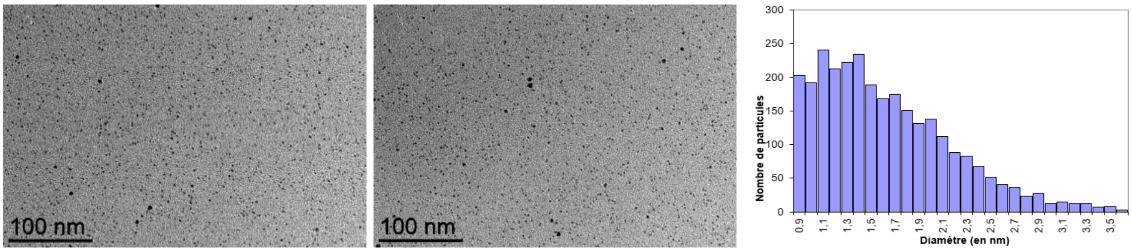

Figure S3. TEM micrographs and size distribution (nm) of the PVP-Pd NPs immobilized in glycerol ether **100**.
Medium size: 1.4 ± 0.6 nm (for 3609 particles).

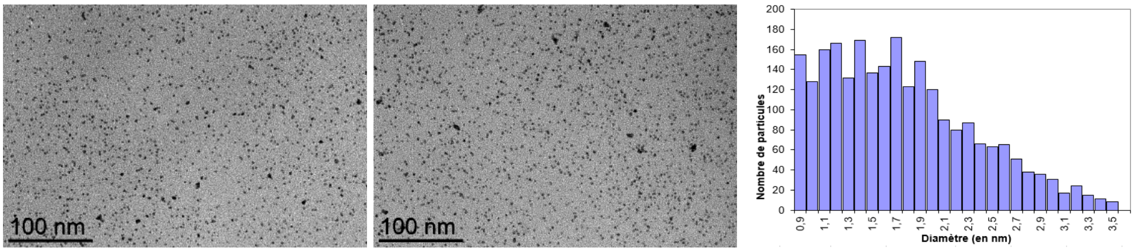

Figure S4. TEM micrographs and size distribution (nm) of the PVP-Pd NPs immobilized in glycerol ether **200**.
Medium size: 1.5 ± 0.7 nm (for 2994 particles).

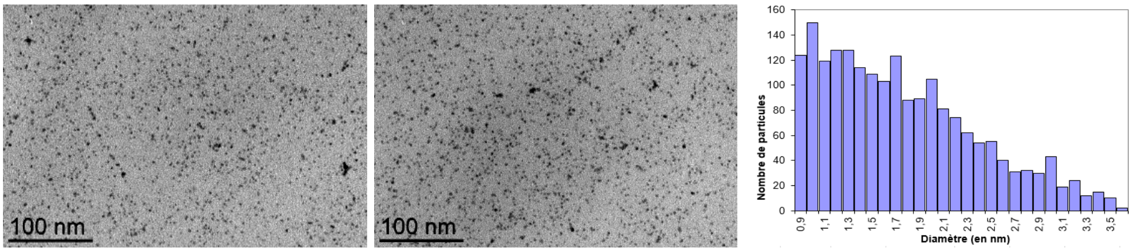

Figure S5. TEM micrographs and size distribution (nm) of the PVP-Pd NPs immobilized in glycerol ether **3i00**.

Medium size: 1.5 ± 0.7 nm (for 2465 particles).

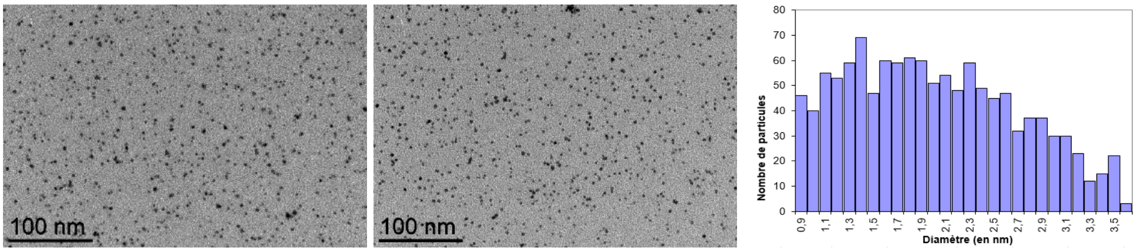

Figure S6. TEM micrographs and size distribution (nm) of the PVP-Pd NPs immobilized in glycerol ether **3F00**.

Medium size: 1.8 ± 0.8 nm (for 1401 particles).

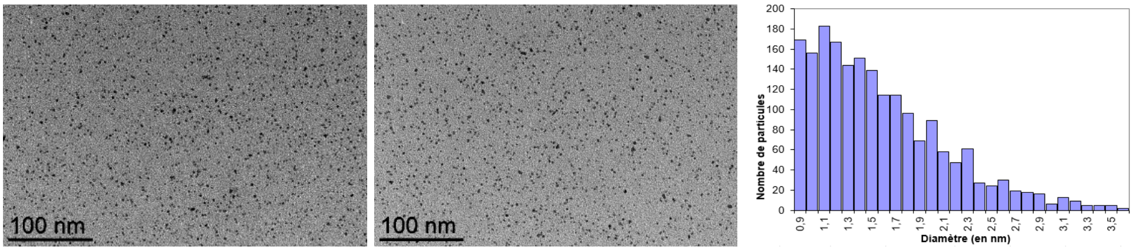

Figure S7. TEM micrographs and size distribution (nm) of the PVP-Pd NPs immobilized in glycerol ether **3F03F**.

Medium size: 1.3 ± 0.6 nm (for 2710 particles).

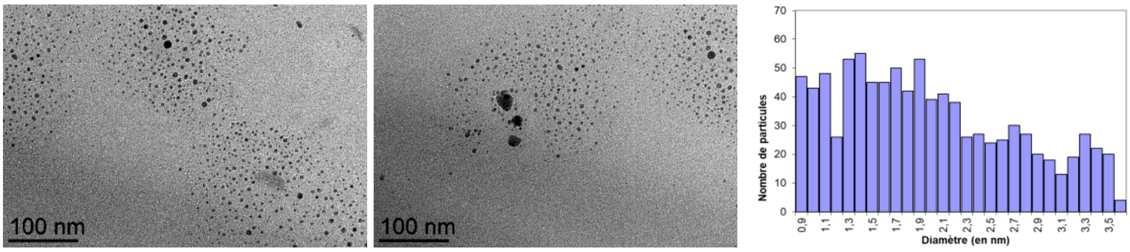

Figure S8. TEM micrographs and size distribution (in nm) of the Pd NPs immobilized in the solvent **N00Cl-000**.

Medium size: 1.7 ± 0.8 nm (for 1137 particles).

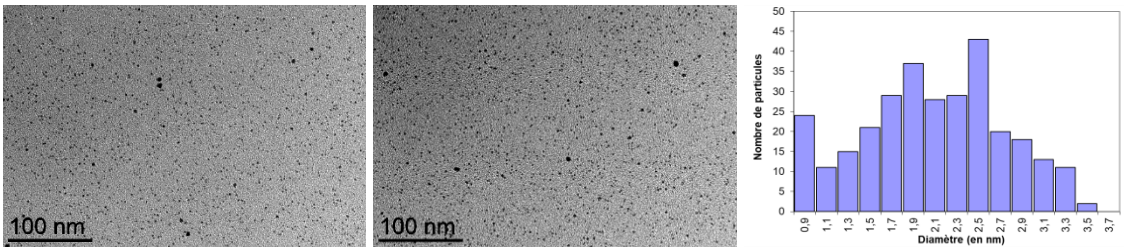

Figure S9. TEM micrographs and size distribution (in nm) of the Pd NPs immobilized in the solvent **N00Cl-100**.

Medium size: 1.8 ± 0.9 nm (for 397 particles).

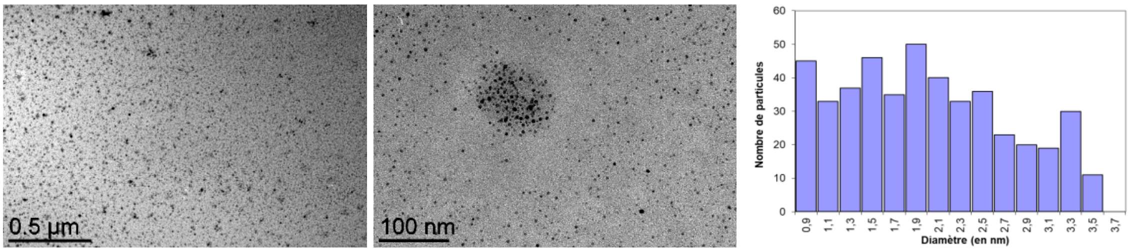

Figure S10. TEM micrographs and size distribution (in nm) of the Pd NPs immobilized in the solvent **N00Cl-3i00**.
Medium size: 1.9 ± 0.9 nm (for 525 particles).

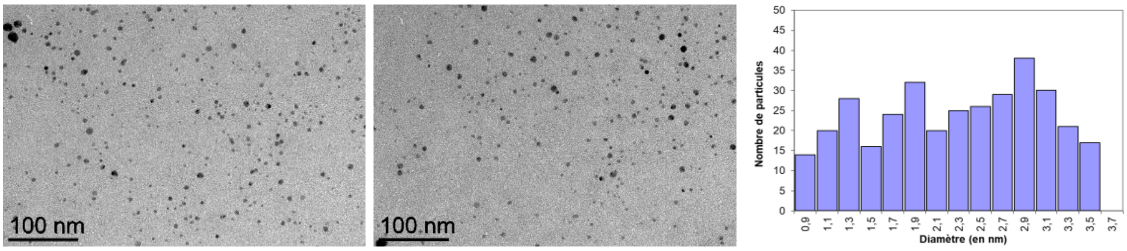

Figure S11. TEM micrographs and size distribution (in nm) of the Pd NPs immobilized in the solvent **N00Cl-3F00**.
Medium size: 2.2 ± 0.9 nm (for 394 particles).

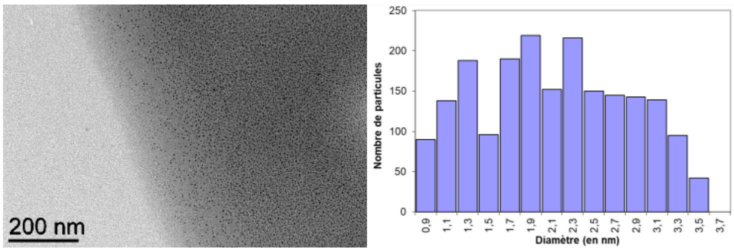

Figure S12. TEM micrographs and size distribution (nm) of the Pd NPs immobilized in solvent **N00Cl-100-3F03F**.
Medium size: 2.0 ± 0.8 nm (for 2226 particles).

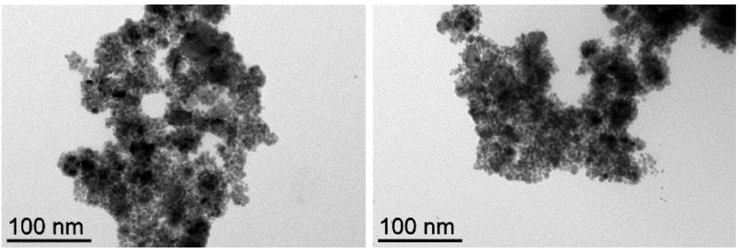

Figure S13. TEM micrographs of the Pd NPs immobilized in the solvent **ChCl-000**.
Medium size: Not available.

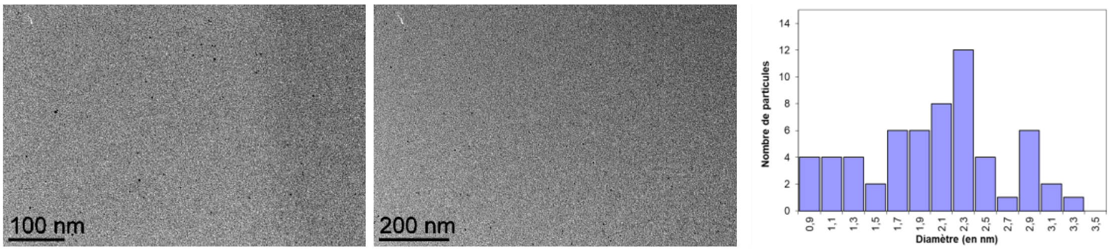

Figure S14. TEM micrographs and size distribution (in nm) of the Pd NPs immobilized in the solvent **ChCl-100**.
Medium size: 1.7 ± 0.8 nm (for 800 particles).

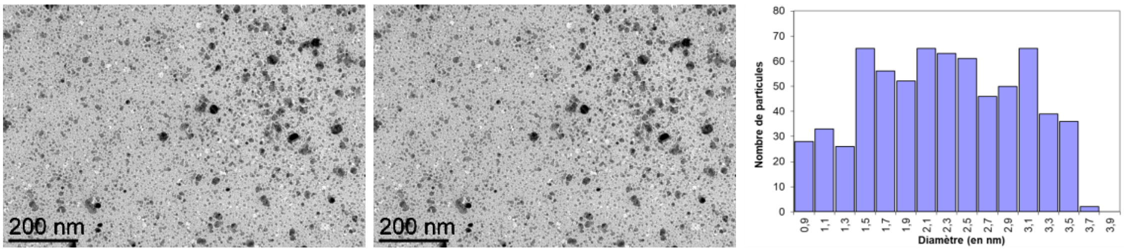

Figure S15. TEM micrographs and size distribution (in nm) of the Pd NPs immobilized in the solvent **ChCl-3F00**. Medium size: 2.2 ± 0.8 nm (for 761 particles).

4.2. – Full characterization of system Pd NPs/N00Cl-100

The catalytic system **Pd NPs/N00Cl-100** was fully characterized both in liquid phase and at solid state. TEM micrographs in liquid phase were obtained with a JEOL JEM 1400 microscope running at 120 kV. IR spectra at solid state were registered in a Varian 640 FTIR spectrometer in the range of 4000–400 cm^{-1}. Elemental and ICP-AES analyses were performed in the "Service d'Analyse of Laboratoire de Chimie de Coordination" of Toulouse, using, respectively, a Perkin Elmer 2400 Series II analyzer and an iCAP 6300 ICP spectrometer. XPS analyses were carried out at "Institut Català de Nanociència i Nanotecnologia" (ICN2, Barcelona, Spain) with a SPECS PHOIBOS 150 hemispherical analyzer (SPECS GmbH, Berlin, Germany) at room temperature in a base pressure of $5{\cdot}10^{-10}$ mbar and using monochromatic Al K_{α} radiation (1486.74 eV, 350 W) as the excitation source. Finally, powder X-ray diffraction pattern analyses were collected at room temperature on a PANalytical X'Pert MPD Pro (θ-θ) diffractometer using Cu $K_{\alpha 1}$, $K_{\alpha 2}$ radiation [α (Cu $K_{\alpha 1}$, $K_{\alpha 2}$) = 1.54060 and 1.54443 Å].

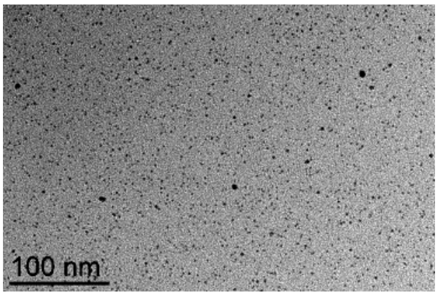

Figure S16. TEM micrograph of fresh Pd NPs immobilized in the solvent **N00Cl-100** in liquid phase.

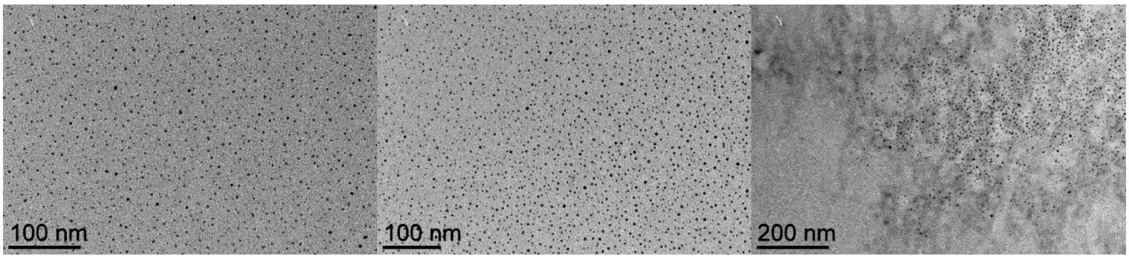

Figure S17. TEM micrographs of the Pd NPs immobilized in solvent **N00Cl-100** after 1, 2 and 6 reaction cycles. Medium sizes: 2.2 ± 1.3 nm (3615 particles); 2.2 ± 1.3 nm (3128 particles); 2.1 ± 0.8 nm (671 particles).

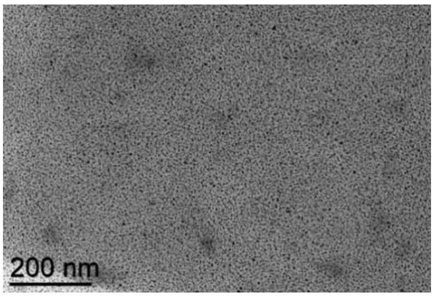

Figure S18. TEM micrograph of Pd NPs immobilized in **N00Cl-100** at solid state.

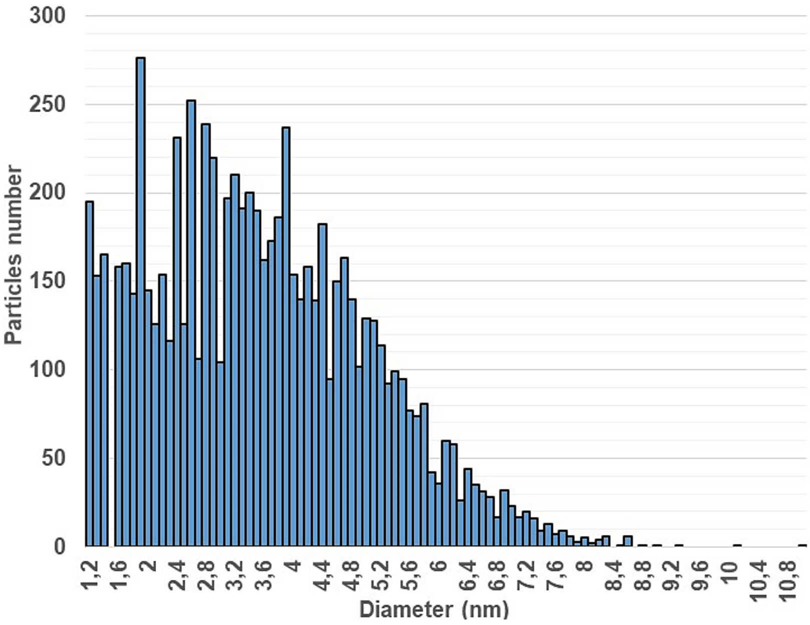

Figure S19. Size distribution of the Pd NPs immobilized in the solvent **N00Cl-100** at solid state. Medium size: 3.3 ± 2.1 nm (for 7688 particles).

Table S1. Results of elemental (EA), ICP and XPS analyses of the system N00Cl-100 at solid state.

Element	EA (%)	ICP (%)	XPS (%)	XPS (wt%)
Carbon	5.29	5.23	29.3	10.9
Hydrogen	0.46	0.39	-	-
Nitrogen	0.95	0.97	2.8	1.2
Chlorine	-	-	1.3	3.2
Oxygen	-	-	48.1	23.8
Palladium	88.9	88.0 [a]	18.5	60.9

[a] Determined by ICP-AES spectroscopy.

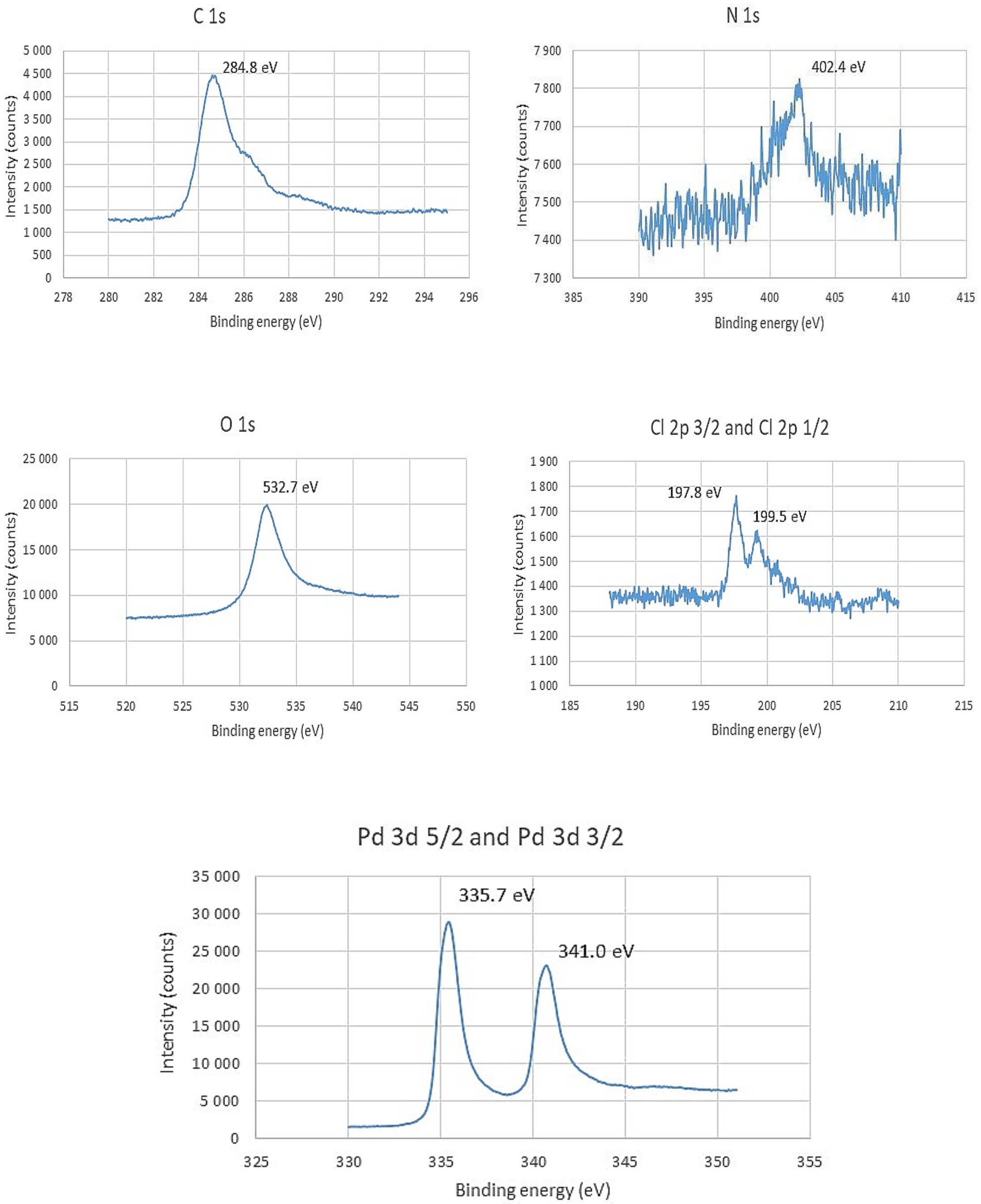

Figure S20. High Resolution XPS spectra of the system **Pd NPs/N00Cl-100** at solid state for carbon (C), nitrogen(N), oxygen (O), chlorine (Cl), and Pd(0).

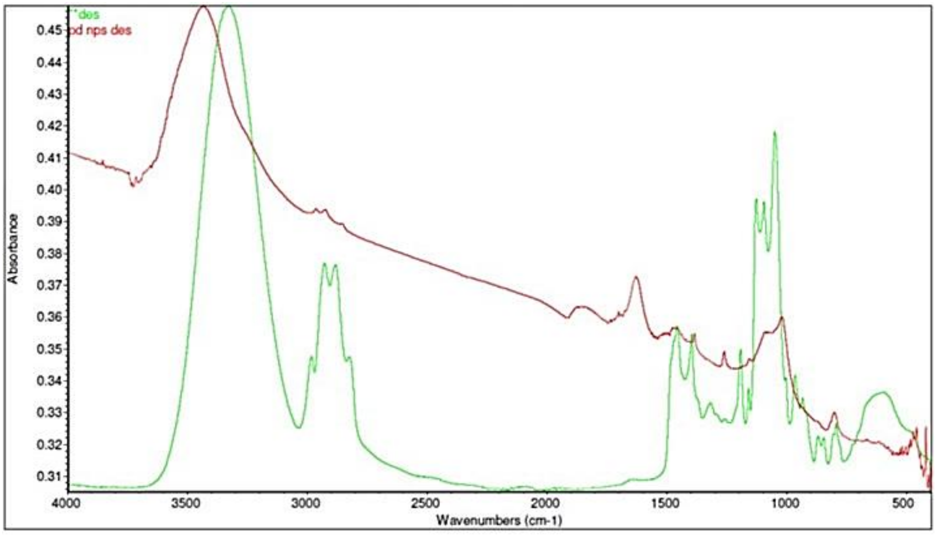

Figure S21. Superposition of the FT-IR spectra of the catalytic system **Pd NPs/N00Cl-100** at solid state (in red color) and that of pure solvent **N00Cl-100** (in green color).

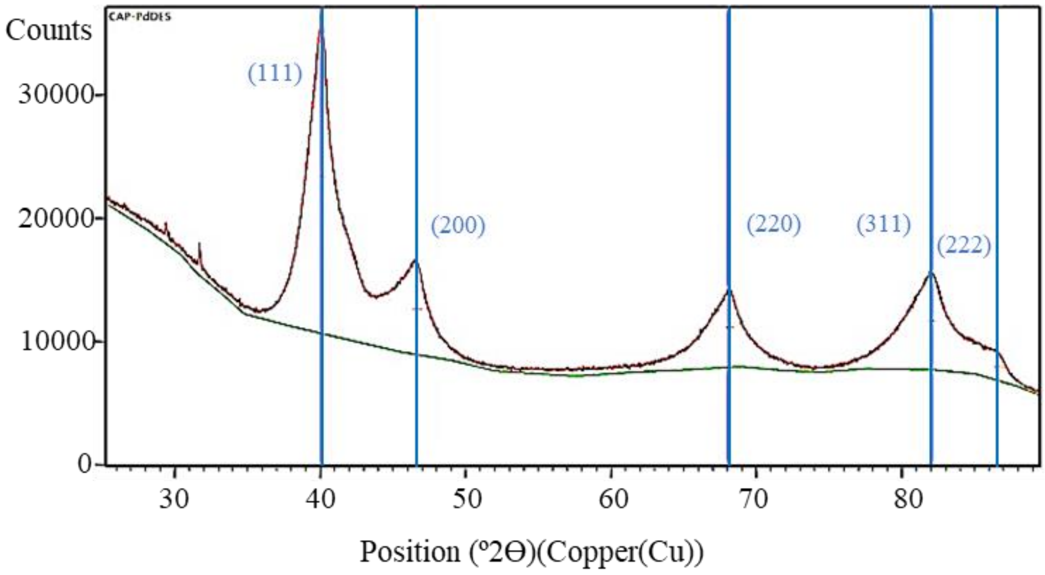

Pos.[°2θ]	Height	d-spacing[Å]	Rel.Int.[%]
40.1327	25955.54	2.24692	100.00
46.6835	7585.39	1.94575	29.22
68.1227	6553.51	1.37648	25.25
82.0302	8097.77	1.17475	31.20
86.6355	2373.02	1.12374	9.14

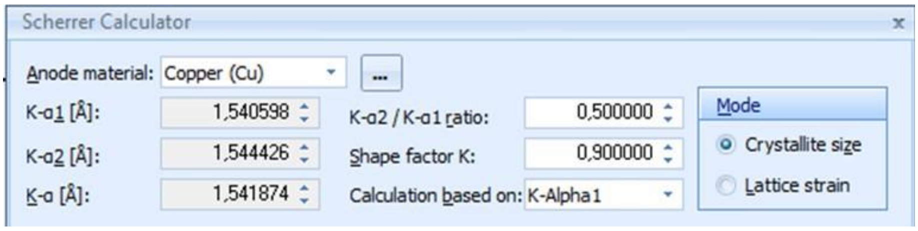

Figure S22. X-ray powder diffractogram of crystalline Pd(0) in system **Pd NPs/N00Cl-100**.

4.3. – ^{1}H NMR spectra of DES after hydrogenation

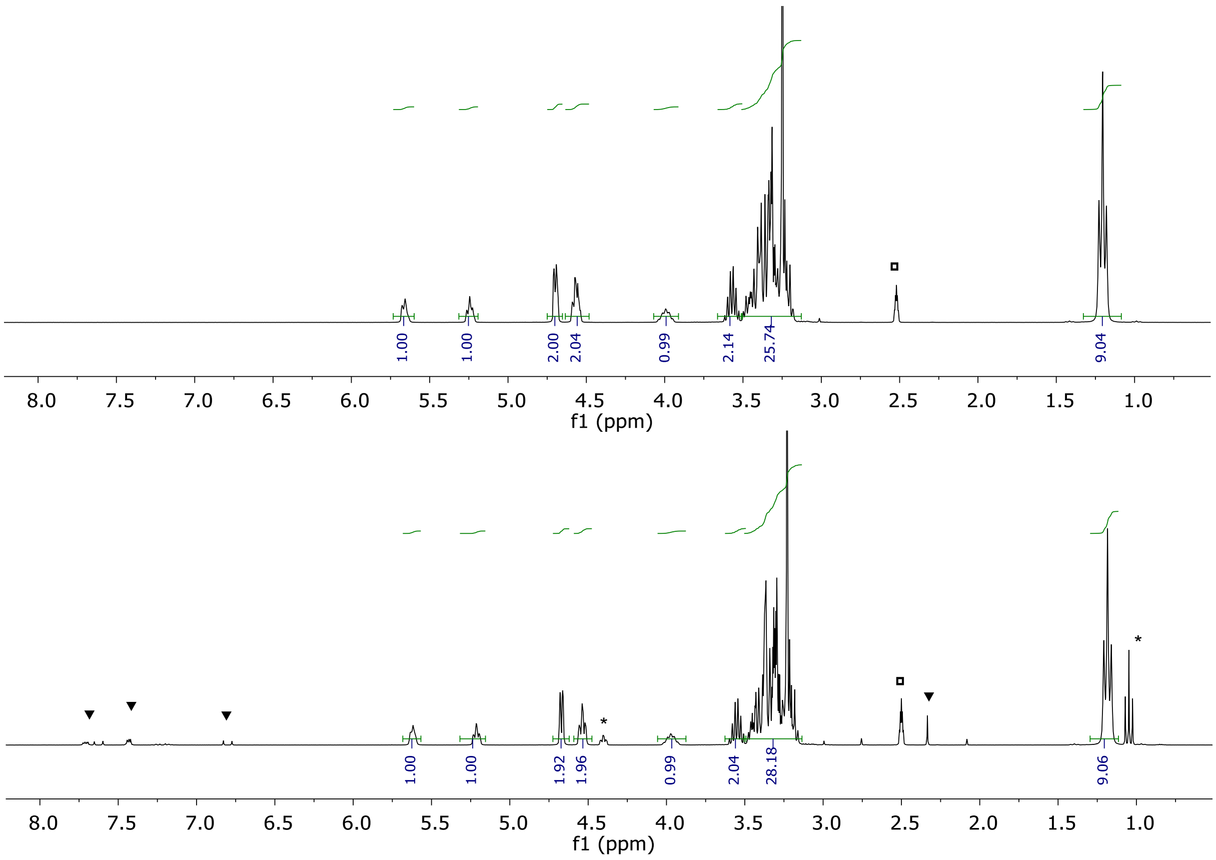

Figure S23. ^{1}H NMR (300 MHz, DMSO-d_6) spectra of **N00Cl-100**: neat (top) and after recycling (bottom).
*Note: * denotes ethanol signals; ▼ denotes signals of residual **1** and **1H**; ◻ denotes signals of DMSO.*

5. - Characterization of hydrogenation products

Hydrogenation products were purified by column chromatography in silicagel or by Kugelrohr distillation, and then characterized by ^{1}H NMR, ^{13}C NMR and GC-MS, in order to compare to literature reports to confirm their spectral identity. NMR spectra (in $CDCl_3$, δ ppm, *J* in Hz) were obtained using a Bruker Avance 300 MHz spectrometer (300 MHz for ^{1}H NMR, and 75.5 MHz for ^{13}C NMR). Mass spectra were acquired using a Perkin Elmer Clarus MS 560 mass detector.

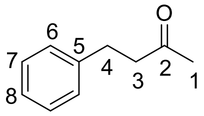

4-Phenylbutan-2-one [1H]: colorless liquid, t_R = 7.45 min. **^{1}H NMR** (300 MHz, $CDCl_3$, 25 ºC): δ 7.30-7.35 (m, 2H, H^7), 7.20-7.25 (m, 3H, H^6, H^8), 2.93 (t, 2H, *J* = 7.5 Hz, H^4), 2.79 (t, 2H, *J* = 7.5 Hz, H^3), 2.17 (s, 3H, H^1). **^{13}C NMR** (75.5 MHz, $CDCl_3$, 25 ºC): δ 208.1 (C_q, C^2), 141.0 (C_q, C^5), 128.5 (CH, C^7), 128.3 (CH, C^6), 126.2 (CH, C^8), 45.2 (CH_2, C^3), 30.1 (CH_3, C^1), 29.8 (CH_2, C^4). **GC-MS** (EI) *m/z*: 149, 148 [$M^{+\cdot}$, 100%], 133, 105, 91, 77, 65, 43.

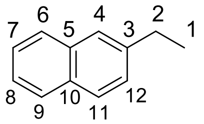

2-Ethylnaphthalene [2H]: colorless liquid, t_R = 8.62 min. **^{1}H NMR** (300 MHz, $CDCl_3$, 25 ºC): δ 7.84-7.93 (m, 3H, H^6, H^9, H^{11}), 7.70-7.74 (m, 1H, H^7), 7.47-7.58 (m, 2H, H^4, H^8), 7.45 (dd, 1H, J_o = 6.0 Hz, J_m = 3.0 Hz, H^{12}), 2.92 (q, 2H, *J* = 7.0 Hz, H^2), 1.43 (t, 3H, *J* = 7.0 Hz, H^1). **^{13}C NMR** (75.5 MHz, $CDCl_3$, 25 ºC): δ 141.8 (C_q, C^3), 133.8 (C_q, C^5), 132.0 (C_q, C^{10}), 127.9 (CH, C^{12}), 127.7 (CH, C^9), 127.5 (CH, C^6), 127.2 (CH, C^{11}), 125.9 (CH, C^7), 125.6 (CH, C^4), 125.1 (CH, C^8), 29.1 (CH_2, C^2), 15.6 (CH_3, C^1). **GC-MS** (EI) *m/z*: 157, 156 [$M^{+\cdot}$], 142, 141 (100%), 128, 115.

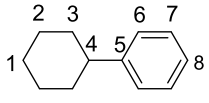

Cyclohexylbenzene [3H]: colorless liquid, t_R = 8.07 min. **^{1}H NMR** (300 MHz, $CDCl_3$, 25 ºC): δ 7.38-7.44 (m, 2H, H^7), 7.22-7.36 (m, 3H, H^6, H^8), 2.52-2.59 (m, 1H, H^4), 1.70-2.00 (m, 4H, $H^{3/3'}$), 1.29-1.55 (m, 6H, $H^{1/1'}$, $H^{2/2'}$). **^{13}C NMR** (75.5 MHz, $CDCl_3$, 25 ºC): δ 148.1 (C_q, C^5), 128.3 (CH, C^7), 126.9 (CH, C^6), 125.8 (CH, C^8), 44.7 (CH, C^4), 34.5 (CH_2, C^3), 27.0 (CH_2, C^1), 26.3 (CH_2, C^2). **GC-MS** (EI) *m/z*: 161, 160 [$M^{+\cdot}$], 131, 117, 115, 104 (100%), 91, 78.

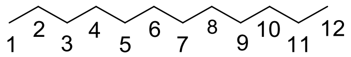

***n*-Dodecane [4H]**: colorless liquid, t_R = 7.00 min. **^{1}H NMR** (300 MHz, $CDCl_3$, 25 ºC): δ 1.25-1.48 (m, 20H, H^2, H^3, H^4, H^5, H^6, H^7, H^8, H^9, H^{10}, H^{11}), 0.94 (t, 6H, *J* = 7.0 Hz, H^1, H^{12}). **^{13}C NMR** (75.5 MHz, $CDCl_3$, 25 ºC): δ 32.0 (CH_2, C^3, C^{10}), 29.8 (CH_2, C^5, C^8), 29.7 (CH_2, C^6, C^7), 29.4 (CH_2, C^4, C^9), 22.7 (CH_2, C^2, C^{11}), 14.1 (CH_3, C^1, C^{12}). **GC-MS** (EI) *m/z*: 171, 170 [$M^{+\cdot}$], 85, 71, 57 (100%), 43, 41.

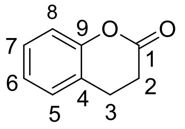

Chroman-2-one [5H]: white solid, t_R = 8.57 min. **^{1}H NMR** (300 MHz, $CDCl_3$, 25 ºC): δ 7.47-7.54 (m, 1H, H^8), 7.00-7.30 (m, 3H, H^5, H^6, H^7), 2.99 (t, 2H, *J* = 7.5 Hz, H^3), 2.78 (t, 2H, *J* = 7.5 Hz, H^2). **^{13}C NMR** (75.5 MHz, $CDCl_3$, 25 ºC): δ 160.8 (C_q, C^1), 154.0 (C_q, C^9), 131.9 (CH, C^5), 128.0 (CH, C^7), 124.5 (CH, C^6), 118.8 (C_q, C^4), 116.7 (CH, C^8), 29.2 (CH_2, C^2), 23.7 (CH_2, C^3). **GC-MS** (EI) *m/z*: 149, 148 [$M^{+\cdot}$, 100%], 120, 119, 91, 78, 63, 32.

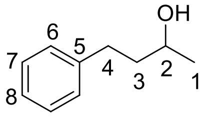

4-Phenylbutan-2-ol [1OH]: colorless liquid, t_R = 7.65 min. **^{1}H NMR** (300 MHz, $CDCl_3$, 25 ºC): δ 7.30-7.36 (m, 2H, H^7), 7.21-7.25 (m, 3H, H^6, H^8), 4.29-4.42 (m, 1H, H^2), 2.94 (t, 2H, *J* = 7.5 Hz, H^4), 1.96-2.08 (m, 2H, H^3), 1.28 (d, 3H, *J* ≈ 9 Hz, H^1). **^{13}C NMR** (75.5 MHz, $CDCl_3$, 25 ºC): δ 141.8 (C_q, C^5), 128.5 (CH, C^7), 128.2 (CH, C^6), 125.8 (CH, C^8), 67.1 (CH, C^2), 40.8 (CH_2, C^4), 32.0 (CH_2, C^3), 24.0 (CH_3, C^1). **GC-MS** (EI) *m/z*: 151, 150 [$M^{+\cdot}$], 132, 117 (100%), 92, 91, 78, 65, 45.

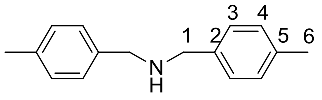

Bis(4-methylbenzyl)amine: t_R = 11.96 min. **^{1}H NMR** (300 MHz, $CDCl_3$, 25 ºC): δ 7.15-7.27 (m, 8H, H^3, H^4), 3.79 (s, 4H, H^1), 2.37 (s, 6H, H^6). **^{13}C NMR** (75.5 MHz, $CDCl_3$, 25 ºC): δ 137.5 (C_q, C^2), 136.9 (C_q, C^5), 129.9 (CH, C^3), 129.1 (CH, C^4), 52.8 (CH_2, C^1), 21.1 (CH_3, C^6). **GC-MS** (EI) *m/z*: 226, 225 [$M^{+\cdot}$], 224, 120, 105 (100%), 91, 77.

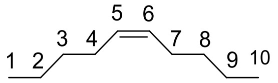

***(Z)*-5-Decene [6H]**: colorless liquid, t_R = 5.64 min. **^{1}H NMR** (300 MHz, $CDCl_3$, 25 °C): δ 5.39 (t, 2H, *J* = 6.0 Hz, H^5, H^6), 2.06 (dt, 4H, *J* = 7.5 Hz, *J* = 6.0 Hz, H^4, H^7), 1.30-1.42 (m, 8H, H^2, H^3, H^8, H^9), 0.94 (t, 6H, *J* = 7.5 Hz, H^1, H^{10}). **^{13}C NMR** (75.5 MHz, $CDCl_3$, 25 °C): δ 129.9 (CH, C^5, C^6), 32.0 (CH_2, C^4, C^7), 26.9 (CH_2, C^3, C^8), 22.4 (CH_2, C^2, C^9), 14.0 (CH_3, C^1, C^{10}). **GC-MS** (EI) *m/z*: 141, 140 [$M^{+\cdot}$], 83, 70, 69, 56, 55 (100%), 41.

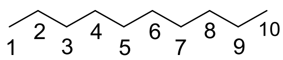

***n*-Decane [6S]**: colorless liquid, t_R = 5.20 min. **^{1}H NMR** (300 MHz, $CDCl_3$, 25 °C): δ 1.29-1.42 (m, 16H, H^2, H^3, H^4, H^5, H^6, H^7, H^8, H^9), 0.94 (t, 6H, *J* = 7.5 Hz, H^1, H^{10}). **^{13}C NMR** (75.5 MHz, $CDCl_3$, 25 °C): δ 32.0 (CH_2, C^3, C^8), 29.7 (CH_2, C^5, C^6), 29.4 (CH_2, C^4, C^7), 22.7 (CH_2, C^2, C^9), 14.0 (CH_3, C^1, C^{10}). **GC-MS** (EI) *m/z*: 142 [$M^{+\cdot}$], 113, 99, 85, 71, 57 (100%), 56, 43, 41, 32.

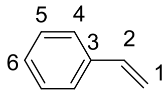

Styrene [7H]: colorless liquid, t_R = 4.06 min. **^{1}H NMR** (300 MHz, $CDCl_3$, 25 °C): δ 7.34-7.43 (m, 2H, H^5), 7.24-7.32 (m, 3H, H^4, H^6), 6.81 (dd, 1H, J_{trans} = 18.0 Hz, J_{cis} = 10.0 Hz, H^2), 5.84 (dd, 1H, J_{trans} = 18.0 Hz, J_{gem} = 1.5 Hz, H^{1trans}), 5.33 (dd, 1H, J_{cis} = 10.0 Hz, J_{gem} = 3.0 Hz, H^{1cis}). **^{13}C NMR** (75.5 MHz, $CDCl_3$, 25 °C): δ 137.6 (C_q, C^3), 137.0 (CH, C^2), 128.6 (CH, C^5), 128.4 (CH, C^4), 126.3 (CH, C^6), 113.9 (CH_2, C^1). **GC-MS** (EI) *m/z*: 105, 104 [$M^{+\cdot}$, 100%], 103, 102, 78, 77, 51.

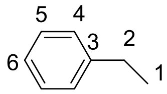

Ethylbenzene [7S]: colorless liquid, t_R = 3.71 min. **^{1}H NMR** (300 MHz, $CDCl_3$, 25 °C): δ 7.39-7.44 (m, 2H, H^5), 7.28-7.34 (m, 3H, H^4, H^6), 2.78 (q, 2H, *J* = 7.5 Hz, H^2), 1.37 (t, 3H, *J* = 7.5 Hz, H^1). **^{13}C NMR** (75.5 MHz, $CDCl_3$, 25 °C): δ 144.3 (C_q, C^3), 128.4 (CH, C^5), 128.0 (CH, C^4), 125.7 (CH, C^6), 29.0 (CH_2, C^2), 15.7 (CH_3, C^1). **GC-MS** (EI) *m/z*: 107, 106 [$M^{+\cdot}$], 91 (100%), 78, 65, 51.

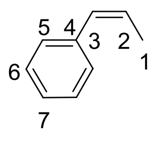

1-Phenyl-1-*(Z)*-propene [8H]: colorless liquid, t_R = 5.11 min. **^{1}H NMR** (300 MHz, $CDCl_3$, 25 °C): δ 7.47-7.50 (m, 2H, H^5), 7.31-7.39 (m, 3H, H^6, H^7), 6.54 (dd, 1H, J_{cis} = 12.0 Hz, *J* ≈ 1.5 Hz, H^3), 5.89 (dq, 1H, J_{cis} = 12.0 Hz, J_{vec} = 7.5 Hz, H^2), 1.99 (d, 3H, J_{vec} = 7.5 Hz, H_1). **^{13}C NMR** (75.5 MHz, $CDCl_3$, 25 °C): δ 137.7 (C_q, C^4), 130.0 (CH, C^3), 128.9 (CH, C^6), 128.2 (CH, C^5), 126.8 (CH, C^7), 126.5 (CH, C^2), 14.6 (CH_3, C^1). **GC-MS** (EI) *m/z*: 119, 118 [$M^{+\cdot}$], 117 (100%), 115, 91, 58.

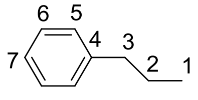

Propylbenzene [8S]: colorless liquid, t_R = 4.71 min. **^{1}H NMR** (300 MHz, $CDCl_3$, 25 °C): δ 7.40-7.45 (m, 2H, H^6), 7.29-7.34 (m, 3H, H^5, H^7), 2.74 (t, 2H, *J* = 7.5 Hz, H^3), 1.80 (sext, 2H, *J* = 7.5 Hz, H^2), 1.10 (t, 3H, *J* = 7.5 Hz, H^1). **^{13}C NMR** (75.5 MHz, $CDCl_3$, 25 °C): δ 142.8 (C_q, C^4), 128.5 (CH, C^6), 128.3 (CH, C^5), 125.7 (CH, C^7), 38.2 (CH_2, C^3), 24.7 (CH_2, C^2), 13.9 (CH_3, C^1). **GC-MS** (EI) *m/z*: 121, 120 [$M^{+\cdot}$], 92, 91 (100%), 78, 65.

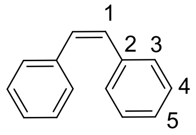

***(Z)*-1,2-Diphenylethene,** or **cis-stilbene [9H]**: white solid; t_R = 9.53 min. **^{1}H NMR** (300 MHz, $CDCl_3$, 25 °C): δ 7.32-7.70 (m, 10H, H^3, H^4, H^5), 6.76 (s, 2H, H^1). **^{13}C NMR** (75.5 MHz, $CDCl_3$, 25 °C): δ 137.4 (C_q, C^2), 130.4 (CH, C^4), 129.1 (CH, C^3), 128.4 (CH, C^5), 127.3 (CH, C^1). **GC-MS** (EI) *m/z*: 181, 180 [$M^{+\cdot}$], 179 (100%), 178, 165, 152.

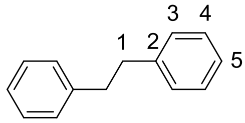

1,2-Diphenylethane, or **bibenzyl [9S]**: white solid; t_R = 9.52 min. **^{1}H NMR** (300 MHz, $CDCl_3$, 25 °C): δ 7.32-7.37 (m, 4H, H^4), 7.23-7.30 (m, 6H, H^3, H^5), 2.99 (s, 4H, H^1). **^{13}C NMR** (75.5 MHz, $CDCl_3$, 25 °C): δ 141.8 (C_q, C^2), 128.5 (CH, C^4), 128.4 (CH, C^3), 126.0 (CH, C^5), 38.0 (CH_2, C^1). **GC-MS** (EI) *m/z*: 183, 182 [$M^{+\cdot}$], 165, 91 (100%), 65.

6. - H_2-mediated hydrodehalogenation results

Hydrodehalogenation reactions can be a hydrogen-mediated catalytic process with interest in the remediation of halogenated organic compounds, such as carcinogenic chlorinated wastes. As a proof of concept, the catalytic system **Pd NPs/N00Cl-100** was applied to the Pd-catalyzed H_2-mediated hydrodehalogenation of aromatic substrates (Scheme S1).

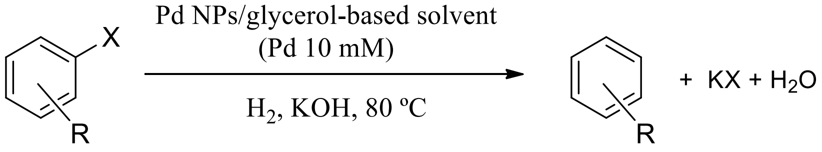

Scheme S1. Pd-catalyzed H_2-mediated hydrodehalogenation of haloaromatics, catalyzed by palladium nanoparticles immobilized in solvent **N00Cl-100**.

After studying the experimental parameters, we concluded the necessity of using catalyst, high hydrogen pressures and potassium hydroxide as base in order to neutralize the formed hydrogen halide. As expected, very low reactivity was observed in the hydrodehalogenation of different aliphatic substrates, such as chlorocyclohexane, bromocyclohexane, 1-chlorodecane or 1-bromodecane. When testing haloaromatic substrates, and using 1 mol% Pd and 20 bar H_2 pressure, *ca.* 20% conversion of 4-chloro-1,2-dimethylbenzene to orthoxylene was observed. If increasing the hydrogen pressure up to 55 bar, conversions of 100% of chlorobenzene to benzene and 78% of 1,3,5-trichlorobenzene to 1,3-dichlorobenzene were achieved, thus broadening the efficiency of these immobilized Pd NPs in different hydrogen-mediated catalytic processes.
